# Activation of Csm6 ribonuclease by cyclic nucleotide binding: in an emergency, twist to open

**DOI:** 10.1093/nar/gkad739

**Published:** 2023-09-25

**Authors:** Stuart McQuarrie, Januka S Athukoralage, Stephen A McMahon, Shirley Graham, Katrin Ackermann, Bela E Bode, Malcolm F White, Tracey M Gloster

**Affiliations:** Biomedical Sciences Research Complex, School of Biology, University of St Andrews, North Haugh, St Andrews, Fife KY16 9ST, UK; Biomedical Sciences Research Complex, School of Biology, University of St Andrews, North Haugh, St Andrews, Fife KY16 9ST, UK; Biomedical Sciences Research Complex, School of Biology, University of St Andrews, North Haugh, St Andrews, Fife KY16 9ST, UK; Biomedical Sciences Research Complex, School of Biology, University of St Andrews, North Haugh, St Andrews, Fife KY16 9ST, UK; Biomedical Sciences Research Complex, School of Chemistry, Centre of Magnetic Resonance, University of St Andrews, North Haugh, St Andrews, Fife KY16 9ST, UK; Biomedical Sciences Research Complex, School of Chemistry, Centre of Magnetic Resonance, University of St Andrews, North Haugh, St Andrews, Fife KY16 9ST, UK; Biomedical Sciences Research Complex, School of Biology, University of St Andrews, North Haugh, St Andrews, Fife KY16 9ST, UK; Biomedical Sciences Research Complex, School of Biology, University of St Andrews, North Haugh, St Andrews, Fife KY16 9ST, UK

## Abstract

Type III CRISPR systems synthesize cyclic oligoadenylate (cOA) second messengers as part of a multi-faceted immune response against invading mobile genetic elements (MGEs). cOA activates non-specific CRISPR ancillary defence nucleases to create a hostile environment for MGE replication. Csm6 ribonucleases bind cOA using a CARF (CRISPR-associated Rossmann Fold) domain, resulting in activation of a fused HEPN (Higher Eukaryotes and Prokaryotes Nucleotide binding) ribonuclease domain. Csm6 enzymes are widely used in a new generation of diagnostic assays for the detection of specific nucleic acid species. However, the activation mechanism is not fully understood. Here we characterised the cyclic hexa-adenylate (cA_6_) activated Csm6’ ribonuclease from the industrially important bacterium *Streptococcus thermophilus*. Crystal structures of Csm6’ in the inactive and cA_6_ bound active states illuminate the conformational changes which trigger mRNA destruction. Upon binding of cA_6_, there is a close to 60° rotation between the CARF and HEPN domains, which causes the ‘jaws’ of the HEPN domain to open and reposition active site residues. Key to this transition is the 6H domain, a right-handed solenoid domain connecting the CARF and HEPN domains, which transmits the conformational changes for activation.

## Graphical Abstract

**Graphical Abstract:**
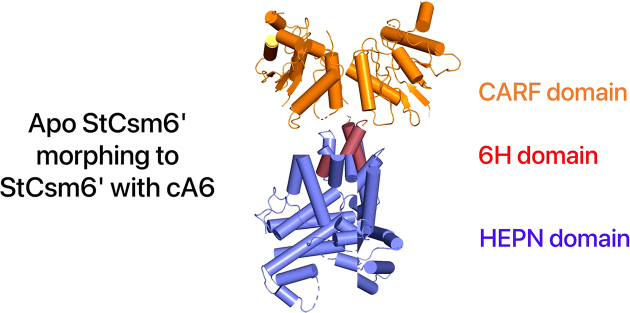

## INTRODUCTION

Type III CRISPR systems eliminate invading nucleic acids via a multi-faceted immune response which, in addition to target RNA and non-specific ssDNA cleavage at the effector complex, involves the activation of ancillary defence enzymes. Target RNA binding and recognition licenses cyclic oligoadenylate (cOA) second messenger synthesis by the large Cas10 subunit of the effector complex (1,2). cOA, composed of 4 or 6 3′-5′ linked AMP subunits (denoted cA_4_ or cA_6_), binds to CARF (CRISPR-associated Rossmann Fold) domains, activating CRISPR ancillary defence nucleases to abrogate viral replication. CARF-family proteins include the ribonucleases Csx1/Csm6 (3–10), PD-D/ExK family DNA nucleases Can1 (11) and Can2/Card1 (12,13) and the transcription regulator Csa3 (14). cA_4_ or cA_6_ stimulate Csx1/Csm6 enzymes by binding to a dimeric CARF domain, which allosterically activates the connected HEPN (Higher Eukaryotes and Prokaryotes Nucleotide-binding) RNase domains for cellular defence. cA_4_ binding to dimeric Card1 results in a rotation of the two monomers with respect to one another, coupled with activation of the nuclease domains (12). In the past few years, the properties of Csx1/Csm6 proteins as activatable, non-specific RNases have been harnessed in a range of diagnostic assays to detect specific nucleic acid species (15–19).

Csx1/Csm6 enzymes cleave nucleic acid non-selectively, therefore collateral damage to host transcripts during the immune response slows cell growth (5), posing a significant risk to long-term cell survival. To remove extant cOA, many type III CRISPR systems encode dedicated ring nucleases of the Crn1-3 families (20,21), which deactivate Csx1/Csm6 enzymes by degrading the antiviral second messenger (reviewed in (22)). Some Csx1/Csm6 enzymes also degrade their own cOA activators (4,23–27) and viruses utilize highly active anti-CRISPR ring nucleases of the DUF1874 (AcrIII-1) family to subvert type III immunity (28).

For historical reasons, CARF-domain containing CRISPR ancillary ribonucleases associated with type III-A systems have been termed Csm6 and those associated with type III-B/D as Csx1. However, this classification does not adequately reflect the structural differences observed across the family. Proteins that recognize a cA_4_ activator are found in both the Csm6 and Csx1 groups (4,6,29). Although the enzymes are obligate dimers, some assemble into hexameric (trimer of dimer) conformations (6).The activation of this sub-class of enzymes on cA_4_ binding appears to be quite subtle, with minimal changes in the architecture of the proteins observed when the apo and cA_4_-bound structures are compared (4,6).

The subset of Csm6 enzymes that recognize cA_6_, with representatives in *Streptococcus thermophilus*, *Mycobacterium tuberculosis* and *Enterococcus italicus*, have a significantly different domain organization, with an alpha-helical ‘6H’ domain linking the CARF and HEPN domains (30). The structure of *E. italicus* Csm6 (EiCsm6) bound to a fluorinated analogue of the cA_6_ activator revealed the activated form of this family (24). cA_6_ binding was predicted to cause conformational changes that activate the HEPN RNase site, while deactivation upon cA_6_ cleavage at the CARF domain would reverse these changes. Unfortunately, the absence of a Csm6 apo structure has prevented a direct test of this hypothesis.

Here we carried out a biochemical characterization of *S. thermophilus* Csm6’ (StCsm6’) and solved X-ray crystal structures in the presence and absence of cA_6_. This revealed a dramatic conformational change upon cA_6_ binding, with a pronounced rotation of the dimeric CARF domains (through 58°), transmitted via the 6H domain, to prise open the HEPN domains, like the opening of a jaw (see Video Abstract). Based on these structures and supporting biochemical and EPR (electron paramagnetic resonance) data, we propose a model for activation of Csm6 proteins by cA_6_, whereby a large rotation of the CARF domains results in activation of the HEPN domains.

## MATERIALS AND METHODS

### Cloning

A synthetic gene (g-block) encoding *S. thermophilus* Csm6’, codon optimized for expression in *Escherichia coli* (Supplementary Figure S1), was purchased from Integrated DNA Technologies (IDT), Coralville, USA and cloned into the pEV5HisTEV vector (31) between the *Nco*I and *Bam*HI restriction sites. The variants H336A, R331E, S105W, D80Y, (C223A:C292A:N209C:R85C) and (C223A:C292A:N209C:D88C), were generated using the QuikChange Site-Directed Mutagenesis kit as per manufacturer's instructions (Agilent Technologies).

### Expression and purification of Csm6’ and variants

The pEV5HisTEV-*csm6’* wild-type and mutant constructs were transformed into C43 (DE3) *E. coli* cells. StCsm6’ protein was expressed according to the standard protocol described previously (Rouillon et al., 2019). Briefly, 2–4 l of cell culture (in LB broth) containing the pEV5HisTEV-*csm6’* plasmid was induced with 0.4 mM isopropyl-β-D-1-thiogalactoside (IPTG) at an OD_600_ of ∼0.6 and grown overnight at 16°C. Cells were harvested (4000 rpm; Beckman Coulter JLA-8.1 rotor) and resuspended in lysis buffer containing 50 mM Tris–HCl pH 7.5, 0.5 M NaCl, 10 mM imidazole and 10% glycerol. Cells were lysed by sonicating six times, with cycles of 1 min on ice and 1 min rest intervals and the cell debris removed by centrifugation. StCsm6’ was purified with a 5 ml HisTrapFF column (GE Healthcare); following loading of the supernatant following cell lysis, the column was washed with 20 column volumes (CV) of buffer containing 50 mM Tris–HCl pH 7.5, 0.5 M NaCl, 30 mM imidazole and 10% glycerol, and the protein was eluted with a linear gradient of buffer containing 50 mM Tris–HCl pH 7.5, 0.5 M NaCl, 0.5 M imidazole and 10% glycerol across 15 CV. Protein containing fractions were concentrated and the 8-His affinity tag was removed by incubating protein with Tobacco Etch Virus (TEV) protease (10:1 StCsm6’:TEV protease) overnight at room temperature.

Cleaved StCsm6’ was further purified by repeating the immobilized metal affinity chromatography step and collecting the unbound fraction. Size exclusion chromatography (HiLoad 16/60 Superdex 200 pg, GE Healthcare) was used to complete purification, with pure StCsm6’ protein eluted isocratically in buffer containing 20 mM Tris–HCl pH 7.5, 150 mM NaCl. The protein was concentrated using a centrifugal concentrator, aliquoted and frozen at –70°C.

For seleno-methionine labelled expression, the plasmid containing the *csm6’* gene was transformed into *E. coli* B834 (DE3) cells. Cells were grown in M9 minimal medium supplemented with Selenomethionine Nutrient Mix (Molecular Dimensions, Newmarket, Suffolk, UK) and 50 mg l^−1^ (l)-selenomethionine (Acros Organics). The protein was purified by the same method described for native StCsm6’. StCsm6’ variants were expressed and purified as described for the WT protein.

### Radiolabelled RNA cleavage assays

To determine RNA cleavage by StCsm6’ and variants, 50 nM radiolabelled RNA oligonucleotide A1 (5′AGGGUAUUAUUUGUUUGUUUCUUCUAAACUAUAAGCUAGUUCUGGAGA) was incubated with protein (1 μM dimer) and cOA activator (cA_3_, cA_4_ or cA_6_) (BIOLOG, Life Sciences Institute, Bremen) in buffer containing 20 mM HEPES pH 7.0, 150 mM NaCl, 1 mM dithiothreitol (DTT), 1 mM EDTA and 3 units SUPERase·In RNase inhibitor at 45°C. Control reactions incubating RNA in buffer without protein, and RNA with protein in the absence of cOA activator were also carried out. Reactions were stopped by adding phenol-chloroform and vortexing to remove protein, and 5 μl of reaction product was extracted into 5 μl 100% formamide xylene-cyanol loading dye. All experiments were carried out in triplicate and RNA cleavage was visualized by phosphor imaging following denaturing polyacrylamide gel electrophoresis (PAGE).

For deactivation assays, cA_6_ (10, 20, 50, 100, 200 nM) was preincubated with buffer alone (control) or with StCsm6’, S105W or H336A variants (1 μM dimer) for 60 min at 45°C prior to adding 50 nM radiolabelled RNA and StCsm6’ (1 μM dimer) and incubating for a further 30 min at 45°C. Control reactions, with cOA not preincubated or treated with StCsm6’ (c1), and no cOA but RNA and StCsm6’ added after preincubation of buffer (c2), were carried out. Reactions were stopped by adding phenol-chloroform as detailed above and RNA cleavage was visualized by phosphor imaging after denaturing PAGE. RNA cleavage was quantified using the Bio-Formats plugin (32) of ImageJ as distributed in the Fiji package (33). RNA protected was calculated using the no cOA control reaction as 100% protection. All experiments were carried out in triplicate.

### Fluorogenic RNA cleavage assays

StCsm6’ was incubated with a doubling series of cA_6_ activator (0.02, 0.03, 0.06, 0.12, 0.24, 0.49, 0.98, 1.95, 3.91, 7.81, 15.63, 31.25, 62.5, 125, 250 nM) and 125 nM RNaseAlert FAM™ reporter substrate (IDT) in a 30 μl volume in buffer containing 20 mM MES pH 6.0, 30 mM NaCl and 20% glycerol for 30 min at 45°C. Fluorescence intensity (excitation wavelength at 490 nm and emission at 520 nm) corresponding to RNA cleavage was measured during this period using a FLUOstar Omega microplate reader (BMG LABTECH) with gain set to 2100. Experiments were carried out in triplicate.

### Crystallization

StCsm6’ was mixed with cA_6_ (hereafter StCsm6’-cA_6_) in a 1:1.5 molar ratio of StCsm6’:cA_6_ and incubated at room temperature for 30 min prior to crystallization. Crystallization trials were performed using sitting drop vapour diffusion, with JCSG and PACT 96-well commercial screens (Jena Bioscience), set up with an Art Robbins Gryphon robot. Non-labelled StCsm6’-cA_6_ crystallized at a concentration of 12.5 mg/ml and the selenomethionine-labelled StCsm6’-cA_6_ (hereafter SeMet-StCsm6’-cA_6_) at 10 mg/ml. Following optimization using hanging drops in a 24 well plate, crystals were obtained from 1.85 M sodium malonate and 5% ethanol, over a reservoir volume of 600 μl, for StCsm6’-cA_6_, and 1.6 M sodium malonate, over a reservoir volume of 1 ml, for SeMet-StCsm6’-cA_6_. 3 μl drops in a 2:1 or 1:1 protein:mother liquor ratio were added to a silanized cover slip and sealed with high-vacuum grease (DOW Corning, USA) and incubated at room temperature. Crystals were harvested and cryoprotected with the addition of 20% PEG 1000 and 10% glycerol to the mother liquor, mounted on loops and vitrified in liquid nitrogen.

No diffraction quality crystals were immediately forthcoming for apo StCsm6’; a crystal was observed after 6–12 months incubation in the initial 96 well crystallization screens, with the drop noticeably dehydrated. Apo-StCsm6’was crystallized from 0.95 M sodium citrate, 0.18 M sodium bromide and 0.1 M HEPES, pH 8. Crystals were harvested and cryoprotected with the addition of 20% PEG 1000 to the mother liquor, mounted on loops and vitrified in liquid nitrogen.

### X-ray data processing, structure solution and refinement

X-ray data for SeMet-StCsm6’-cA_6_ were collected at Diamond Light Source (DLS) on beamline I04, at a wavelength of 0.9795 Å, to 2.61 Å resolution. Diffraction data were automatically processed through the Xia2 pipeline (34), using XDS and XSCALE (35), and showed a strong anomalous signal. The data were phased using the automated experimental phasing pipeline SHELX (36) in CCP4 Online (37), and an initial model of StCsm6’ was built using the ARP/wARP webservice (38). No further refinement was carried out on this model.

X-ray data for StCsm6’-cA_6_ were collected at DLS on beamline I04 and processed to 1.96 Å resolution using autoPROC (39) and STARANISO (40; http://staraniso.globalphasing.org/cgi-bin/staraniso.cgi). MOLREP (41) was used to phase the data for StCsm6’-cA_6_ using molecular replacement with the model generated by ARP/wARP (from the SeMet-StCsm6’-cA_6_ data) as the search model. Iterative cycles of REFMAC5 (42) or PHENIX (43) and COOT (44) were used for automated and manual refinement of the model respectively, including addition of water molecules. Electron density for cA_6_ was clearly visible in the maximum likelihood/σA weighted *F*_obs_ – *F*_calc_ electron density map at 3σ. cA_6_ was drawn using Chemdraw (Perkin Elmer), restraints generated in JLigand (45), and positioned using COOT (44).

X-ray data for apo-StCsm6’ were collected at DLS on beamline I03 and processed to 3.54 Å resolution using FAST_DP (34), which incorporates XDS (35), CCP4 (37) and CCTBX (46). Molecular replacement using PHASER (47) was used to phase the apo-StCsm6’ data, using the StCsm6’-cA_6_ structure as the model (with cA_6_ removed). Successful phasing required the N- and C-terminal domains of StCsm6’ to be separated into two distinct search models. The model was refined as described above.

Throughout refinement, the quality of both models was assessed and validated using PDB-Redo (48) and Molprobity (49), and corrected as required. For StCsm6’-cA_6_, the Molprobity score is 1.35; 99th centile. Ramachandran statistics are 97% favoured; 2% allowed; 1% outliers. For apo-StCsm6’, the Molprobity Clashscore is 3.14; 84th centile. Ramachandran statistics are 76% favoured; 18% allowed; 6% outliers. Data processing and refinement statistics are shown in Supplementary Table 1. Structural figures and movies were created using CCP4MG (50) and PyMol (Schrödinger Inc.). Alignments were performed using the DALI server (51), and all values calculated are shown in Supplementary Tables 2 and 3. The model coordinates and structure factors have been deposited in the Protein Data Bank with accession codes 8PE3 (StCsm6’ in complex with cA_6_) and 8PCW (apo-StCsm6’).

### Spin labelling

Purified protein (200 μl, ∼250 μM) of StCsm6’ variants C223A:C292A:N209C:R85C (hereafter R85C/N209C) and C223A:C292A:N209C:D88C (hereafter D88C/N209C) in 20 mM Tris, 150 mM NaCl, pH 7.5, were incubated overnight at 4°C with DTT at a final concentration of 7.5 mM to freshly reduce cysteine residues. DTT was removed using a desalting column (PD10, Cytiva). Eluted protein solutions were concentrated to a volume of 1.5–2 mL and incubated with a 20-fold molar excess (10-fold with respect to cysteine residues) of *S*-[(1-oxyl-2,2,5,5-tetramethyl-2,5-dihydro-1H-pyrrol-3-yl)methyl] methanesulfonothioate (MTSL) spin label (Santa Cruz Biotechnology) overnight at 4°C, yielding the spin-labelled side chain R1. Excess MTSL was removed using a PD10 column.

Successful spin labelling was confirmed via electrospray ionization (ESI) mass spectrometry using the in-house mass spectrometry facility. ESI mass spectrometry was performed on samples before (control) and after spin labelling. Samples were diluted to 1 μM in 1% formic acid (FA). 10 μl per sample was injected onto the liquid chromatography (LC) system (Waters Xevo G2 TOF MS with Acquity HPLC) using a MassPrep cartridge column (Waters), applying a 5 minute gradient from 95% water, 5% acetonitrile to 5% water, 95% acetonitrile (eluents supplemented with 1% FA). Data were collected in positive mode from 500–2500 m/z and charged ion series deconvolution to 0.1 Da resolution was performed using the MaxEnt I algorithm utilizing a peak width at half height of 0.4 *m*/*z*. Expected masses were obtained for both mutants before and after labelling (see Supplementary Figure S2). Labelling efficiencies were obtained from continuous wave (CW) EPR spectra as described below (Supplementary Figure S2).

### Sample preparation for pulse dipolar EPR spectroscopy and cryogenic CW EPR

Exchange of the spin labelled protein into deuterated buffer was performed by means of repeated steps of dilution followed by centrifugal concentration until a theoretical deuteration of at least 99.5% was reached. Samples for cryogenic CW and pulse EPR measurements were prepared at a final protein concentration of 50 μM (100 μM spin label). Both StCsm6’ mutants were prepared with and without 25 μM cA_6_. 50% (v/v) deuterated glycerol (CortecNet) was used for cryoprotection. The samples with a final volume of 65 μl were transferred to 3 mm quartz EPR tubes which were immediately frozen in liquid nitrogen.

### Pulse dipolar EPR spectroscopy (PDS)

PDS experiments were performed at 50 K on a Bruker ELEXSYS E580 spectrometer with an overcoupled 3 mm cylindrical resonator (ER 5106QT-2w), operating at Q-band frequency (34 GHz), using a second frequency option (E580-400U). Pulses were amplified by a pulse travelling wave tube (TWT) amplifier (Applied Systems Engineering) with nominal output of 150 W. Temperature was controlled using a cryogen-free variable temperature cryostat (Cryogenic Ltd) operating in the 3.5 to 300 K temperature range.

Pulse electron-electron double resonance (PELDOR) experiments were performed with the 4-pulse DEER (52–54) pulse sequence (π/2(ν_A_) – τ_1_ – π(ν_A_) – (τ_1_ + t) – π(ν_B_) – (τ_2_ – t) – π(ν_A_) – τ_2_ – echo) as described previously (55), with a frequency offset (pump – detection frequency) of +80 MHz (∼3 mT). Shot repetition times (SRT) were set to 2 or 3 ms; τ_1_ was set to 380 ns, and τ_2_ was set to 8000 ns for the samples without addition of cA_6_ and to 6500 ns for those with cA_6_. The echo decays as function of available dipolar evolution time were assessed from refocused echo decays by incrementing τ_2_ in the 4 pulse DEER sequence from a start value of 760 ns and omitting the ν_B_ inversion pulse. Pulse lengths were 16 and 32 ns for π/2 and π detection, and 12 or 14 ns for the ELDOR π pump pulse. The pump pulse was placed on the resonance frequency of the resonator and applied to the maximum of the nitroxide field-swept spectrum.

PDS experiments were analyzed using DeerAnalysis2015 (56). PDS data were first background-corrected using a 3D homogeneous background function and ghost suppression (power-scaling) (57) for a four-spin system, before Tikhonov regularization followed by statistical analysis using the validation tool in DeerAnalysis2015, varying background start from 5% to 80% of the trace length in 16 trials. Resulting background start time for the best fit was then used as starting point for a second round of Tikhonov regularization followed by a second round of statistical analysis, this time including the addition of 50% random noise in 50 trials, resulting in a total of 800 trials.

Traces recorded with a τ_2_ of 8000 ns were cut at 7300 for processing to remove artifacts at the end of the trace. Traces recorded with a τ_2_ of 6500 ns resulted in a best fit where the background fit had a positive and thus unphysical slope after the first validation. Therefore, data were cut iteratively by 10% of the initial trace length and Tikhonov regularization and first validation rounds were repeated until the best fit had a decaying background function. This resulted in a 10% cut for the D88R1/N209R1 + cA_6_ data and a 30% cut for the R85R1/N209R1 + cA_6_ data. Validation trials from the second validation round were pruned with a prune level of 1.15, where trials exceeding the root mean square deviation of the best fit by at least 15% were discarded. In all cases the regularization parameter α was chosen according to the L-curve criterion (58) and the goodness-of-fit.

For comparison, raw PDS data were subjected to the ComparativeDEERAnalyzer (CDA) version 2.0 within DeerAnalysis2022 (DEERNet (59) Spinach SVN Rev 5662 and DeerLab (60) 0.9.1 Tikhonov regularization) for user-independent data processing and analysis, in line with current recommendations (61). CDA reports are provided as shown in Supplementary Table 4.

### Continuous wave (CW) EPR

CW EPR measurements were performed using a Bruker EMX 10/12 spectrometer equipped with an ELEXSYS Super Hi-Q resonator at an operating frequency of ∼9.9 GHz (X-band) with 100 kHz modulation.

### Room-temperature CW EPR

Room-temperature CW EPR measurements were performed to assess labelling efficiency. Samples were recorded using a 100 G field sweep centred at 3455 G, a time constant of 20.48 ms, a conversion time of 20.12 ms, and 1707 points resolution. An attenuation of 20.0 dB (2 mW power) and a modulation amplitude of 0.7 G were used. Protonated StCsm6’ samples were measured in 50 μl capillaries at 50 μM protein (100 μM spin) concentration and double integrals were compared to MTSL as a standard. Labelling efficiency was ≥97% for D88R1/N209R1 and ≥87% for R85R1/N209R1, and samples showed negligible free spin label contribution.

### Cryogenic CW EPR

Cryogenic CW EPR spectra were obtained at 120 K. The temperature was controlled with an ER4141 VTM Nitrogen VT unit (Bruker) operated with liquid nitrogen and a quartz Dewar insert. Samples were recorded using a 200 G field sweep centred at 3256 G, a time constant of 20.48 ms, a conversion time of 20.00 ms, and 2000 points resolution. An attenuation of 40.0 dB (20 mW power) and a modulation amplitude of 1 G were used.

To estimate the dipolar broadening by short inter-spin distances in CW EPR, the spectra of cA_6_ bound constructs were simulated as dipolarly broadened spectra obtained by convoluting the apo spectrum with the dipolar broadening functions corresponding to a Gaussian distribution centred at 1.1 and 0.9 nm with widths of 0.2 and 0.35 nm for StCsm6’ R85R1/N209R1 and D88R1/N209R1, respectively.

### Modelling for PDS measurements

Distance distributions were modelled based on the crystal structures obtained in this study for StCsm6’ in the presence and absence of cA_6_. R1 moieties were introduced at residues 85 or 88 and 209 of both chains of the StCsm6’ dimer using mtsslWizard (62) within the mtsslSuite (63) server-based modelling software. Cartoon structural representations of spin-labelled StCsm6’ constructs were generated using Pymol (Schrödinger Inc.).

### Reagents

*Nco*I and *Bam*HI restriction enzymes (New England Biolabs, Hitchin, Herts, UK, cat. no. R3193S and R0136S respectively); QuikChange Site-Directed Mutagenesis kit (Agilent Technologies, Santa Clara, CA 95051, cat. no. 200515); M9 minimal medium supplemented with Selenomethionine Nutrient Mix (Molecular Dimensions, Newmarket, Suffolk, UK, cat. no. MD12-501); (L)-selenomethionine (Acros Organics, Geel, Belgium, cat. no. AC259962500); Tobacco Etch Virus (TEV) protease (recombinantly over-expressed in-house); cA_3_, cA_4_, cA_6_ (BIOLOG, Life Sciences Institute, Bremen, cat. no. C362, C335, C332 respectively); SUPERase·In RNase inhibitor (Thermofisher, Oxford, UK; cat. no. AM2696); 125 nM RNaseAlert FAM™ reporter substrate (Integrated DNA Technologies, Coralville, USA; cat. no. 11–04-02–03); radiolabelled RNA oligonucleotide A1 (5′AGGGUAUUAUUUGUUUGUUUCUUCUAAACUAUAAGCUAGUUCUGGAGA) (Integrated DNA Technologies, Coralville, USA); JCSG and PACT 96-well commercial screens (Jena Bioscience, Jena, Thuringen, Germany, cat. no. CS-206L and CS-207L respectively); Gryphon robot (Art Robbins Instruments, Sunnyvale, CA 94089, USA, cat. no. 620-1020-11); *S*-[(1-oxyl-2,2,5,5-tetramethyl-2,5-dihydro-1H-pyrrol-3-yl)methyl] methanesulfonothioate (MTSL) spin label (Santa Cruz Biotechnology, Dallas, Texas, USA, cat. no. sc-208677); Xevo G2 TOF MS with Acquity HPLC (Waters, Milford, Massachusetts, USA); MassPrep cartridge column (Waters, Milford, Massachusetts, USA, cat. no. 186002785); deuterated glycerol (CortecNet, Les Ulis, France, cat. no. CD1060P10).

### Biological resources

Synthetic gene (g-block) encoding *S. thermophilus* Csm6’, codon optimized for expression in *Escherichia coli* (Integrated DNA Technologies, Coralville, USA); pEHisV5TEV vector (31); *E. coli* C43 (DE3) cells (Sigma Aldrich, Burlington, Massachusetts, USA, cat. no. CMC0019-20 × 40UL); *E. coli* B834 (DE3) cells (Sigma Aldrich, Burlington, Massachusetts, USA, cat. no. 69041–3).

### Statistical analyses

For kinetic analyses, all assays were repeated in triplicate. Means and standard deviations were calculated and shown along with original data points.

### Web sites/data base referencing

Bio-Formats plugin of ImageJ (32), Fiji package (33); Xia2 pipeline (34); XDS and XSCALE (35); SHELX (36); CCP4 Online (37); ARP/wARP webservice (38); autoPROC (39); STARANISO (40); MOLREP (41); REFMAC5 (42); PHENIX (43); COOT (44); Chemdraw (Perkin Elmer); JLigand (45); FAST_DP (34); CCP4 (37); CCTBX (46); PHASER (47); PDB-Redo (48); Molprobity (49); CCP4MG (50); PyMol (Schrödinger Inc.); DALI server (51); DeerAnalysis2015 (56); DeerAnalysis2022: using DEERNet (59) through Spinach SVN Rev 5662 and DeerLab (60) 0.9.1 Tikhonov regularization; mtsslWizard (62); mtsslSuite (63) server.

## RESULTS

### StCsm6’ is a ribonuclease activated by cA_6_

**Figure 1.**
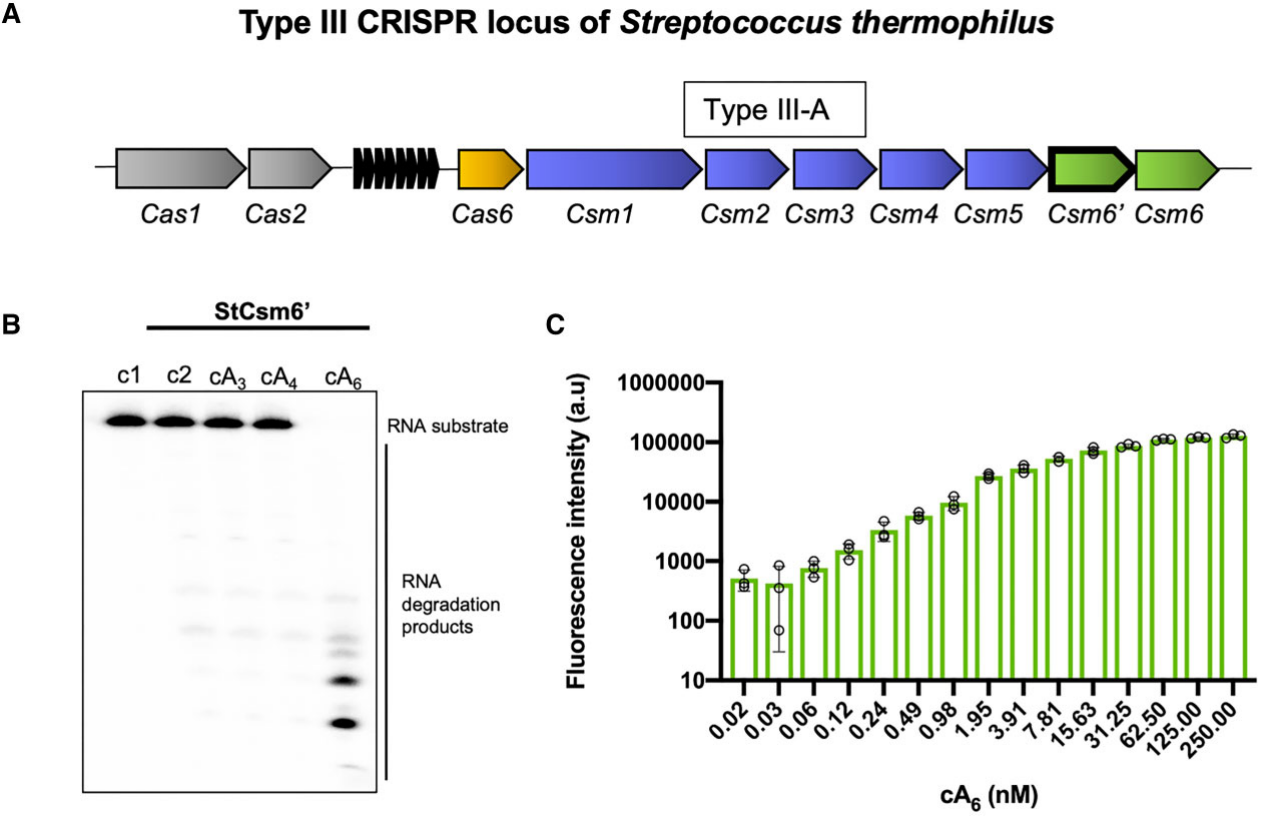
StCsm6’ is a cyclic hexa-adenylate activated ribonuclease. (**A**) Type III CRISPR locus of *Streptococcus thermophilus*. Two cOA activated CARF-family ribonucleases, denoted Csm6 and Csm6’, are found adjacent to *cas* genes encoding the type III CRISPR complex. (**B**) Denaturing PAGE visualizing cleavage of a radiolabelled RNA oligonucleotide A1 by StCsm6’ (1 μM dimer) with cA_3_, cA_4_ or cA_6_ activator (20 μM). StCsm6’ is specifically activated by cA_6_. The image is representative of three technical replicates. Control c1 is RNA alone and c2 is RNA incubated with StCsm6’ in the absence of activator. (**C**) Fluorogenic RNase activity assay; cleavage of a FAM™ reporter substrate (RNaseAlert, IDT) by StCsm6’ (1 μM dimer) across a range of cA_6_ concentrations. Columns depict the mean of three technical replicates (individual data points shown) and error bars show the standard error of the mean.

The type III-A CRISPR system of *S. thermophilus* encodes two Csm6 proteins, denoted Csm6 (StCsm6) and Csm6’ (StCsm6’) (Figure 1A). The kinetic properties of StCsm6 have been studied extensively (25), while StCsm6’ has only been confirmed as a cOA dependent ribonuclease (1). As StCsm6’ was the subject of this study, we first wished to examine its enzymatic properties. We incubated StCsm6’ and radiolabelled RNA with different cOA molecules to confirm that, as expected, StCsm6’ is specifically activated by cA_6_ (Figure 1B). Extending this analysis, we utilized a real-time fluorescence assay to determine the sensitivity to the cA_6_ activator, revealing that StCsm6’ is activated by as little as 60 pM and fully activated by ∼100 nM cA_6_ (Figure 1C).

### Structure of the active form of StCsm6’

**Figure 2.**
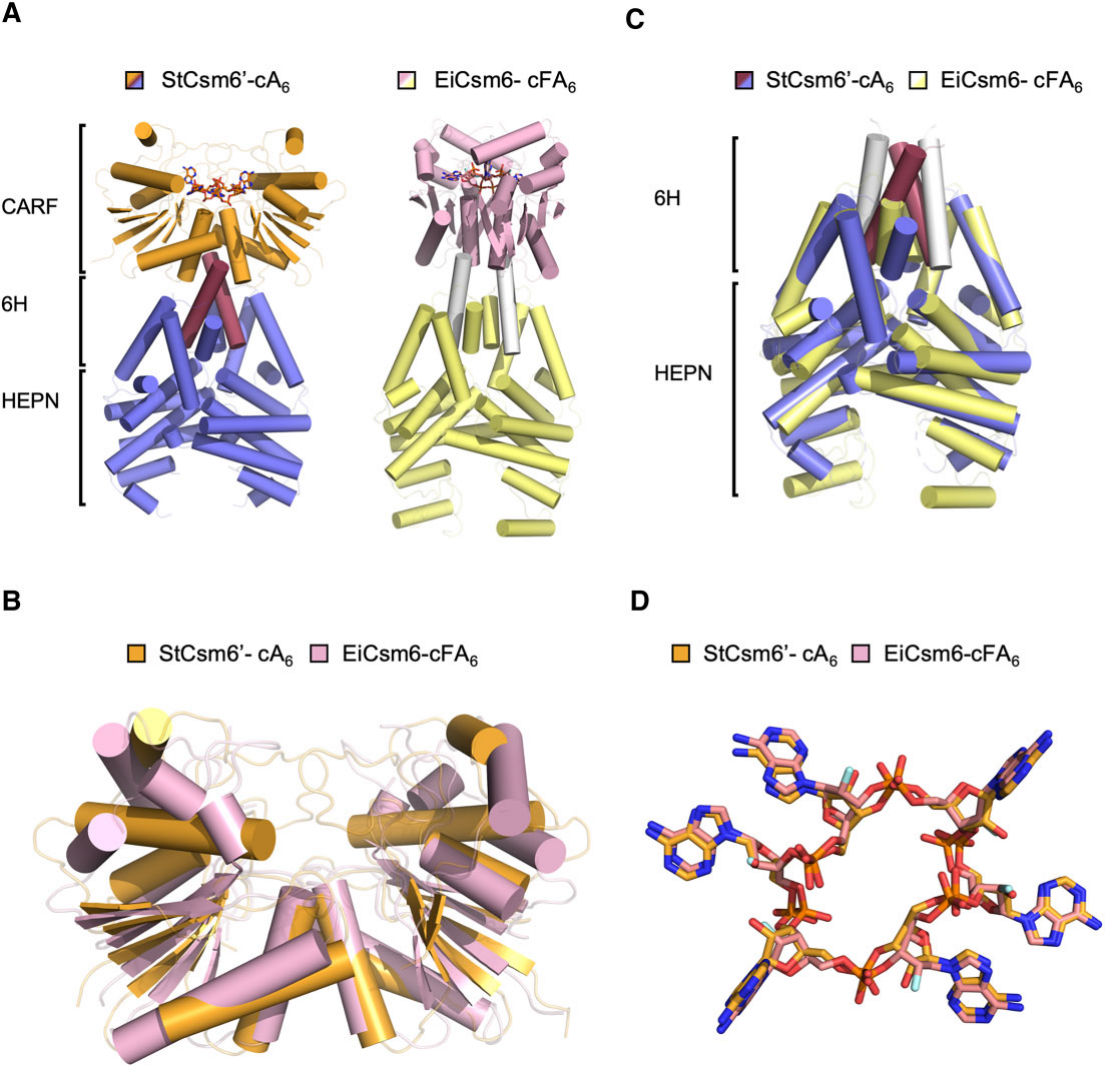
Comparison of StCsm6’ in complex with cA_6_ and EiCsm6 in complex with cFA_6_ structures. (**A**) Cartoon representation of the secondary structure elements of StCsm6’ (left) and EiCsm6 (right); the structures are orientated to give the same view of their HEPN domains. The CARF domains are shown in orange and pink, the 6H and HEPN domains in blue and yellow, and a key α-helix in the 6H domain in burgundy and white for StCsm6’ and EiCsm6, respectively. The cA_6_ molecule bound to StCsm6’ and the cFA_6_ molecule bound to EiCsm6 are shown in stick representation; carbon atoms are shown as orange or pink for StCsm6’ and EiCsm6, respectively, phosphate as dark orange, fluorine as cyan, nitrogen as blue and oxygen as red. (**B**) Structural superimposition of the CARF domains from StCsm6’ and EiCsm6; colours as described in (A). (**C**) Structural superimposition of the 6H and HEPN domains; colours as described in (A). (**D**) Superimposition of the cA_6_ molecule bound to StCsm6’ and cFA_6_ molecule bound to EiCsm6; colours as described in (A).

We proceeded to solve the structure of StCsm6’ bound to its cA_6_ activator (Figure 2A) using X-ray crystallography with data to 1.96 Å resolution. StCsm6’ is symmetrical dimer, with each monomer displaying a CARF domain (residues 1–173), a central 6H domain (residues 174–239) (30), and a HEPN domain (residues 240–386). In the dimer, each domain interacts with the equivalent domain in the other monomer, but they cross over each other at the 6H domains to give an ‘X’ arrangement (Supplementary Figure S3; Supplementary Movie 1). The CARF domain comprises five α-helices and five β-strands which alternate to form a central parallel β-sheet sandwiched between a three helix bundle and the other two helices. The CARF domain is linked to the HEPN domain via the 6H domain, comprising three α-helices (there are a total of six α-helices in the dimer, hence 6H). The HEPN domain comprises a total of six α-helices; the C-terminal helix packs against the α-helices in the 6H domain.

A DALI (51) search shows the closest structural match to StCsm6’ is EiCsm6 in complex with a fluorinated cA_6_ mimic (cFA_6_, where each C2’ hydroxyl group is replaced with a fluorine atom) (PDB: 6TUG) (24), with a root mean square deviation (RMSD) of 6.6 Å over 369 Cα atoms for the monomer; the two proteins have a sequence identity of 26%. Like StCsm6’, EiCsm6 is a dimer, with each monomer possessing a CARF, a 6H and HEPN domain, and displays a similar arrangement where the monomers cross over at the 6H domain. Although the overall RMSD between the two structures is high (and higher still for the dimer, see Supplementary Table 2), this is largely reflective of a different orientation of the domains in the monomer relative to each other. This suggests the possibility of some rotational motion of the CARF domains with respect to the HEPN domains in the dimeric protein.

The monomeric CARF domain of StCsm6’ and EiCsm6 in the cA_6_/cFA_6_ complexes are largely superimposable (RMSD of 2.1 Å over 162 Cα atoms for the monomer) (Figure 2B). The core β-sheet in the CARF domains, as well as the two α-helices closest to the 6H domain, which form the majority of the interactions at the dimer interface, show the highest structural similarity. The region with the three α-helices in the CARF domains of StCsm6’, which are linked by long loops, will require significant movement to ‘open’ in order to allow cA_6_ to bind, and to ‘close’ once cA_6_ is bound. The equivalent region in the CARF domains of EiCsm6, which has four α-helices, one of which is distorted, has a different arrangement of these elements compared to StCsm6’, and thus overall lower structural similarity.

The 6H domain, first predicted bioinformatically (30), links the CARF and HEPN domains. The most structurally similar protein to the StCsm6’ 6H domain is EiCsm6 (RMSD of 1.8 Å over 65 Cα atoms for the monomer), despite only 18% sequence identity. The high structural similarity between these domains in the two proteins, over a relatively small number of residues comprising just three α-helices, suggests this domain plays an important functional role rather than being present solely to link the CARF and HEPN domains.

The closest structural homologues to the HEPN domain of StCsm6’ are EiCsm6 and Csm6 from *Staphylococcus epidermidis* (SeCsm6; PDB code 5YJC) (64), with RMSDs of 1.9 and 1.7 Å, both over 141 Cα atoms of the monomer, respectively. The overall domain structure between the three proteins is very similar (Figure 2C; Supplementary Figure S3B). However, both EiCsm6 and SeCsm6 have an additional ∼40 residues in the HEPN domain compared to StCsm6’. This comprises two short β-strands arranged in an anti-parallel β-sheet, and a 15 residue α-helix close to the tip of the HEPN domain.

### Molecular recognition and cleavage of cA_6_ by StCsm6’

The *F*_obs_-*F*_calc_ map revealed electron density consistent with a molecule of cA_6_ bound to the StCsm6’ dimer, which was fitted into the model (Figure 2A, D). Upon refinement, it became clear there was actually a mix of species in the binding site, which corresponded to intact cA_6_ and the two products of cA_6_ cleavage, linear tri-adenylate molecules containing 2′,3′-cyclic phosphates (A_3_ > P), with occupancies estimated as 0.50 (cA_6_) and 0.50 (both A_3_ > P) (Supplementary Figure S3C). All interactions made with StCsm6’ were the same for both the intact cA_6_ and the hydrolysed products, so will only be described for the former (Figure 2D; Supplementary Figure S4A).

All interactions between cA_6_ and StCsm6’ were made exclusively to residues in the CARF domains, and were symmetrical with respect to each monomer in the dimer. cA_6_ formed hydrogen bonds with main chain atoms in D10, T11, R15, D19, A107, Q110, S133, H136, A137 and N139, and with side chain atoms in T11, S39, D73, K112, N139 and R167, as well as a stacking interaction between H77 and one of its adenosine moieties (Supplementary Figure S4A). cA_6_ is fully enclosed within the binding site of the CARF domains (Supplementary Figure S4B), suggesting there is significant mobility in the region around this site. This is consistent with higher temperature factors displayed by the residues in the α-helix and loops that enclose cA_6_ (Supplementary Figure S4C), which notably is the region least structurally conserved with the CARF domains of EiCsm6.

The presence of both intact cA_6_ and the cleaved A_3_ > P products provides unique insights into catalysis by residues in the CARF domains. Mechanistic studies on cyclic oligoadenylate cleavage propose that an activated 2′-OH group on a ribose is responsible for in-line nucleophilic attack on the adjacent scissile phosphodiester bond (21,24,28). Given the symmetrical nature of the StCsm6’ interactions with cA_6_, cleavage takes place at the identical (but opposite) sides of the cA_6_ ring to produce two A_3_ > P molecules. The observed position of cleavage in cA_6_ is consistent with the notion that the angle formed between the 2′-OH, P and O should be close to 180°. The mean of this angle at the two positions in cA_6_ where cleavage occurs is 172°, compared to 100° and 159° at the other equivalent positions in the cA_6_ ring (Supplementary Figure S5A). Interestingly, the 2′-OH group of ribose and the phosphate involved in cleavage form minimal interactions with StCsm6’; the 2′-OH forms a hydrogen bond with the backbone amide of N10, and an oxygen atom in the phosphate with T11 (Supplementary Figure S5B).

The binding environment for cA_6_ in StCsm6’ is structurally similar to that of EiCsm6 bound to fluorinated cA_6_ (Supplementary Figure S6). cA_6_ in StCsm6’ superimposes with cFA_6_ in EiCsm6 with an RMSD of 1.3 Å over 126 atoms, and visually is conformationally identical (Figure 2D). The conservation of interactions made between the ligands and StCsm6’ or EiCsm6 vary at different positions around the ring. The residues (N10 and T11 in both structures) and hydrogen bond interactions with the 2′-OH of the ribose and phosphate involved in cleavage are absolutely conserved, as are the interactions of the adenine of the same adenylate moiety with main chain atoms of R15 and N19 and side chain of R167. This suggests the arrangement of these residues is critical to getting the cA_6_ into a conformation commensurate with catalysis. The other two pairs of adenylate moieties display very few conserved interactions, with the exception of S39, suggesting a greater plasticity in these regions of the binding site. It is worth noting that overall EiCsm6 makes fewer interactions with cFA_6_ than StCsm6’ with cA_6_.

### Characterization of RNA and cA_6_ cleavage by StCsm6’

**Figure 3.**
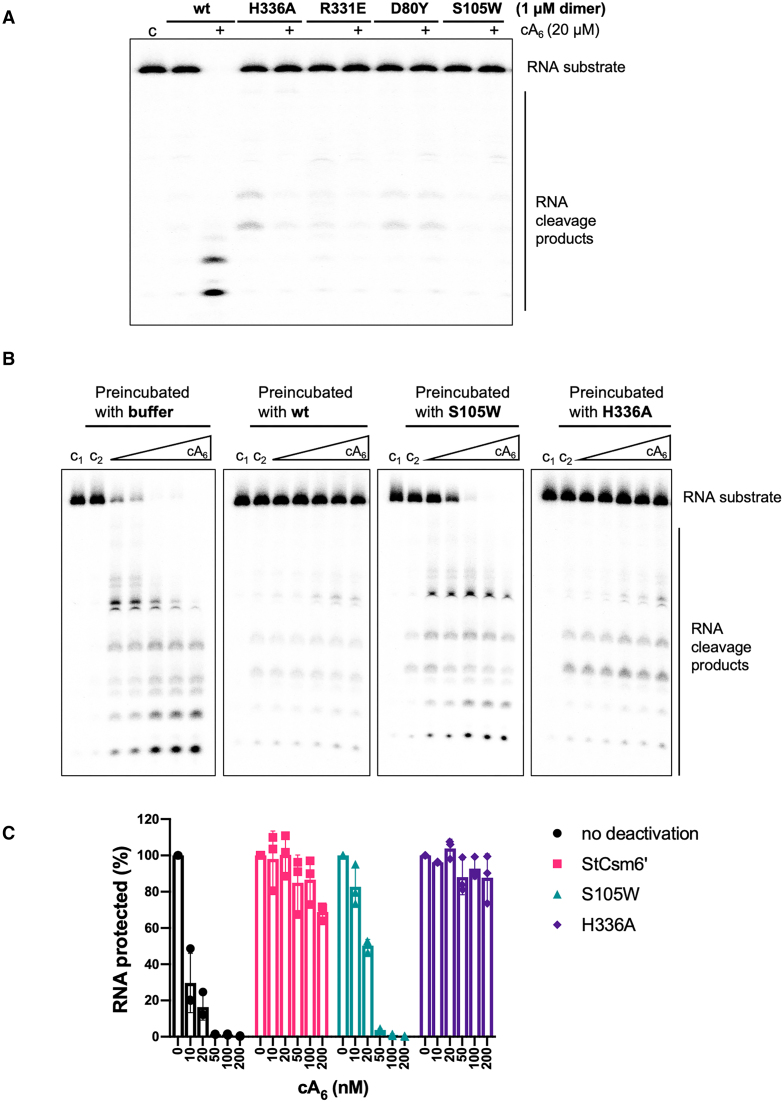
StCsm6’ is a self-limiting ribonuclease which cleaves cA_6_ at the CARF domains. (**A**) Denaturing PAGE gel visualizing RNA A1 cleavage by WT StCsm6’ and variants (all at 1 μM dimer) in the absence and presence (+) of 20 μM cA_6_. Control ‘c’ refers to RNA only; ‘wt’ refers to WT StCsm6’; H336A and R331E are HEPN domain variants; S105W is a CARF domain variant designed to block cA_6_ binding; and D80Y is discussed later. All are catalytically inactive. The image is representative of three technical replicates. (**B**) Deactivation assays where a range of cA_6_ concentrations (10, 20, 50, 100, 200 nM) was incubated with either buffer, WT StCsm6’ (wt), S105W variant or H336A variant (all at 1 μM dimer) at stage 1, prior to addition of radiolabelled RNA A1 and WT StCsm6’ at stage 2, analysed by denaturing PAGE and phosphorimaging. Control ‘c1’ refers to RNA only, not subjected to pre-incubation or enzyme; ‘c2’ refers to no cA_6_ but StCsm6’ and RNA added after buffer incubation. The images are representative of three technical replicates. (**C**) Quantification of the densiometric signals from B to determine the level of RNA protected from WT StCsm6’ activity upon incubating with either buffer, WT StCsm6’, S105W or H336A variants. Bars depict the average from three technical replicates (individual data points shown) and error bars are the standard deviation of the mean.

The mechanism of RNA degradation by StCsm6’ was investigated by site-directed mutagenesis of the arginine and histidine residues within the R-X_4_-H HEPN catalytic motif. We replaced R331 and H336 with glutamate and alanine, respectively (Supplementary Figure S7). Each of these mutations abolished RNA cleavage, as expected (Figure 3A). To verify that cA_6_ binding to the CARF domain was essential for activation of RNA cleavage at the HEPN active site, we generated a S105W variant to disrupt cA_6_ binding at the CARF domain (Supplementary Figure S7). The S105W mutation abolished cA_6_-activated RNA cleavage, demonstrating that replacement of S105 by a bulky tryptophan residue most likely prevents cA_6_ binding and subsequent activation of RNA cleavage by StCsm6’.

Given the observation of a mixed population of cleaved and intact cA_6_ in the crystals, we carried out a two-stage ribonuclease deactivation assay (28) to investigate this biochemically. By pre-incubating StCsm6’ with different concentrations of cA_6_ before adding radiolabelled A1 RNA and fresh wild type (WT) StCsm6’, we could evaluate the extent to which StCsm6’ and variants degraded cA_6_ at stage 1, and thus protected RNA from enzymatic degradation in the second stage of the assay (Figure 3B). We incubated the same range of cA_6_ concentrations with either reaction buffer (control), WT StCsm6’, S105W StCsm6’ (CARF variant) or H336A StCsm6’ (HEPN variant). The control reaction demonstrated that cA_6_ remaining in the reaction mixture after stage 1 could activate RNA degradation by StCsm6’ in stage 2. In contrast, when cA_6_ was pre-incubated with WT StCsm6’, RNA was protected from degradation at stage 2, across the different concentrations of cA_6_ tested. When cA_6_ was preincubated with the H336A variant at stage 1, RNA degradation was greatly reduced at stage 2, suggesting that this variant can still degrade cA_6_ similarly to the WT protein. In contrast, the S105W variant degraded only very small amounts of cA_6_ at stage 1. Thus, the CARF domain seems to be the major site for cA_6_ degradation in StCsm6’ under the conditions tested, which had low (nM) cA_6_ and an excess of enzyme. These data fit well with previous findings for StCsm6 where cA_6_ degradation was measured directly (25). In that study, increasing the cA_6_ concentration to μM levels led to a much greater contribution by the HEPN domain to cA_6_ degradation. Overall, it appears for both enzymes that at low cA_6_ concentrations the activator is degraded by ring nuclease activity in the high affinity CARF domain binding site. This is consistent with our observation of cleaved cA_6_ in the crystal structure.

### Structure of apo StCsm6’ reveals dramatic conformational changes on cA_6_ binding

**Figure 4.**
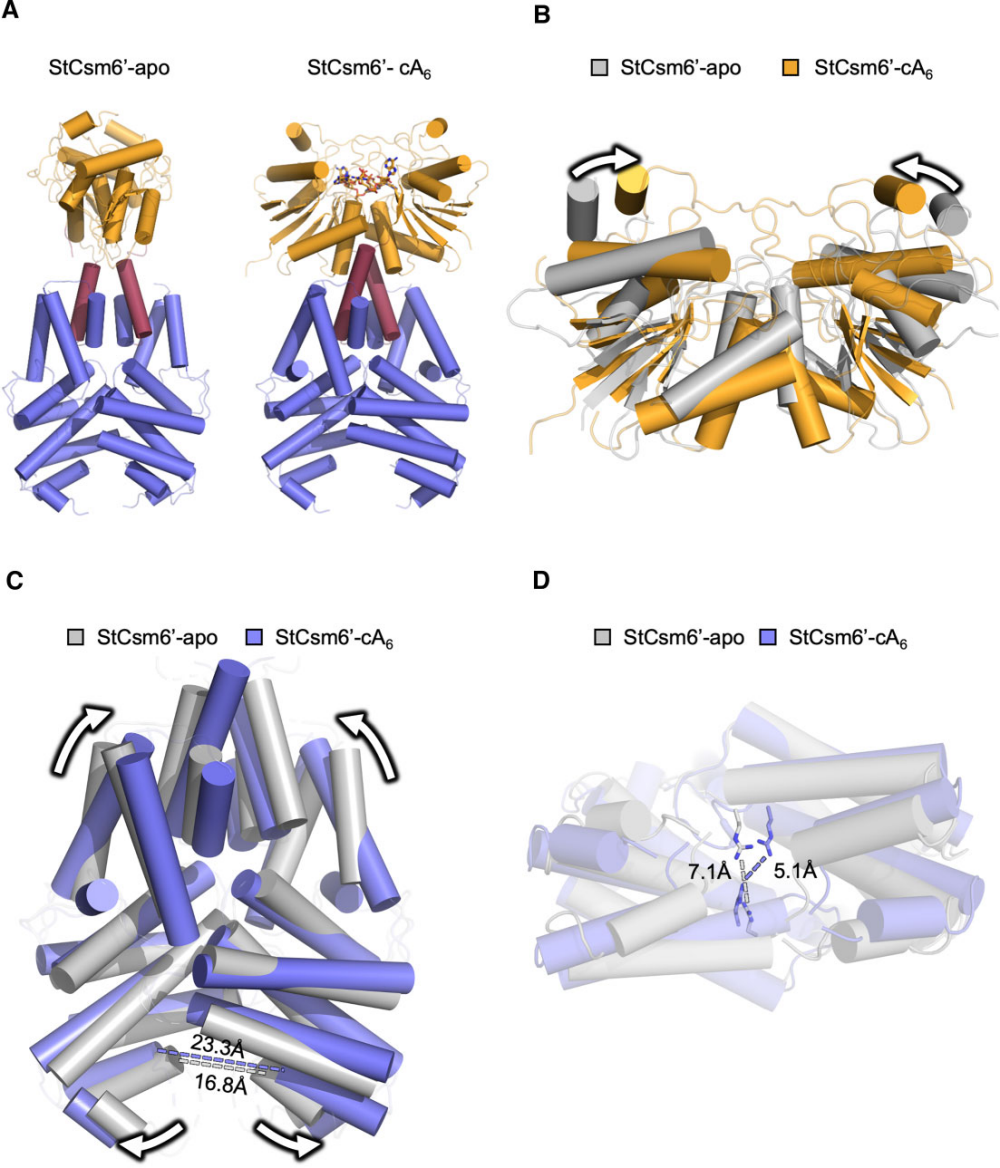
Comparison of the structure of apo StCsm6’ and in complex with cA_6_. (**A**) Cartoon representation of the secondary structure elements of apo StCsm6’ (left) and in complex with cA_6_ (right); the structures are orientated to give the same view of their HEPN domains. The side-by-side view highlights the movement of the CARF domains (orange) relative to the 6H and HEPN domains (blue). A key α-helix in the 6H domain (burgundy) is important in facilitating the movement between the CARF and HEPN domains. The cA_6_ molecule bound to StCsm6’ is shown in stick representation; carbon atoms are shown as orange or pink for StCsm6’ and EiCsm6, respectively, phosphate as dark orange, nitrogen as blue and oxygen as red. (**B**) Superposition of the CARF domains of apo StCsm6’ (grey) and in complex with cA_6_ (orange). The most significant difference is the movement towards the dimer interface of two α-helices which enclose cA_6_, as illustrated by the arrows. (**C**) Superposition of the HEPN domains of apo StCsm6’ (grey) and in complex with cA_6_ (blue). Like the CARF domains, the 6H domains are pulled inwards towards the dimer interface, illustrated by the top arrows. However, the opposite is true for the HEPN domains which swing outwards like the opening of a jaw, illustrated by the bottom arrows. As an example, the distance between F303 (last residue of the α-helix indicated) in each monomer is shown with a dashed line in the colour corresponding to each structure; this shows a movement of 6.5 Å outwards for StCsm6’ in complex with cA_6_ relative to the apo enzyme. (**D**) Superposition of the HEPN domains of apo StCsm6’ (grey) and in complex with cA_6_ (blue), with R331 (first residue in the R-X_4_-H catalytic motif) in each monomer shown in sticks of the same colour. The dashed line, in the colour corresponding to each structure, shows the distance between R331 in each monomer; this highlights a movement of 2.0 Å to bring the arginine residues closer together in the complex with cA_6_, to form a functional active site, relative to the apo enzyme. The other active site residues could not be modelled in the apo structure.

Apo StCsm6’ proved to be extremely difficult to crystallize, and only a single crystal in a dehydrated drop of mother liquor following ∼1 year of incubation was obtained. Consistent with its incalcitrant nature towards crystallization, the resolution of diffraction was low. Nevertheless, data on this crystal were collected to 3.54 Å resolution, and the structure solved by molecular replacement with the separate HEPN and CARF domains of the StCsm6’ complex structure used as the search models. Aside from a couple of disordered regions, the peptide backbone of apo StCsm6’ could be built with confidence, but far fewer side chains were modelled due to the low resolution of the data. The structure revealed that apo StCsm6’, like in the complex with cA_6_, is a symmetrical dimer in an ‘X’ arrangement. Strikingly, however, the overall structure for apo StCsm6’ and in complex with cA_6_ were markedly different, with an RMSD of 7.7 Å over 327 Cα atoms for the monomer and 4.9 Å over 441 Cα atoms for the dimer (note, however, there are 386 residues in the monomer and 772 residues in the dimer, meaning the RMSDs over the full length dimeric protein would be much higher) (Figure 4A). This was evident from the movement of some secondary structure elements in all domains, but in particular the relative orientation of the domains with respect to each other (Supplementary Movie 1).

The monomeric form of the individual CARF, 6H and HEPN domains of StCsm6’ are very similar in the apo structure and in complex with cA_6_, with RMSD values of 1.4 Å or lower over the entirety of each domain (Supplementary Table 3). The only difference to note is that one of the loops in the CARF domain (residues 133–149), which encloses the active site in the complex with cA_6_, could not be modelled in the apo structure, presumably due to conformational flexibility in this region which may not be stabilized until cA_6_ is bound.

However, although the intrinsic secondary structure of individual monomeric domains did not change significantly upon binding of cA_6_, the RMSD values for superimposition of the dimers of each domain were considerably higher (Supplementary Table 3; Supplementary Movie 1). Visual inspection of the superimposition of the apo StCsm6’ and cA_6_ complex structures highlights there was considerable movement in all domains. The most apparent difference in the CARF domains involves an α-helix adjacent to the loops that encloses the cA_6_ binding site, which is outside of the canonical CARF domain core (Figure 4A, B). This α-helix moves up and inwards when in complex with cA_6_. In doing so, there are also some smaller movements in other secondary structure elements in the CARF domains, as a result of the ‘tightening up’ movement towards the dimer interface to both interact with, and enclose, cA_6_. The 6H domain comprises six α-helices (three in each monomer), which are positioned at a similar angle in the two structures, but those in complex with cA_6_ are pulled inwards towards the dimer interface (Figure 4A,C). Whilst upon binding of cA_6_ the CARF and 6H domains appear to tighten with movement towards the dimer interface to enclose cA_6_, at the opposite side of the protein, in the HEPN domain, a number of the secondary structure elements swing outwards to open up the cavity. This is accompanied by a significant rotation of the CARF domains relative to the HEPN domains, of around 58° (Figure 4A, C; Supplementary Figure S8), making the overall effect akin to opening a pair of jaws upon binding of cA_6_ (Supplementary Movie 1, Video Abstract).

The dramatic movement of StCsm6’ upon binding cA_6_ leads to significant changes at the HEPN dimer interface where the catalytic cleavage of RNA takes place. The displacement of the secondary structure elements in the HEPN domain causes an increase in distance of ∼6.5 Å at the opening to the active site (Figure 4C). In turn, this movement brings the catalytic residues closer together in order to form a functional active site. For example, the active site R331 residues of each monomer move closer together by ∼2 Å (Figure 4D; Supplementary Movie 1). Unfortunately, a number of the active site residues could not be modelled in the apo StCsm6’ structure; this could reflect the flexibility in this region prior to cA_6_ binding which forms the functional active site, but also could be a consequence of the lower resolution data.

Unsurprisingly, DALI searches showed that EiCsm6’ was the closest homologue to apo StCsm6’, both over the monomeric full length protein and individual domains (although SeCsm6’ was also a good hit for the HEPN domain) (Supplementary Table 2). As seen with the structure of StCsm6’ in complex with cA_6_, searches with the dimeric units of StCsm6’ generally showed poor alignment with high RMSDs over a limited number of residues. Interestingly, however, the apo StCsm6’ monomer showed a lower RMSD with EiCsm6 in complex with cFA_6_ compared to StCsm6’ in complex with cA_6_ against EiCsm6 in complex with cFA_6_. The RMSDs for the individual domains for both StCsm6’ structures compared to EiCsm6 in complex with cFA_6_ were similar, suggesting that the key difference is the orientation of the domains in relation to each other in each of the full length proteins (Supplementary Figure S9).

### Conformational change probed by pulse EPR

**Figure 5.**
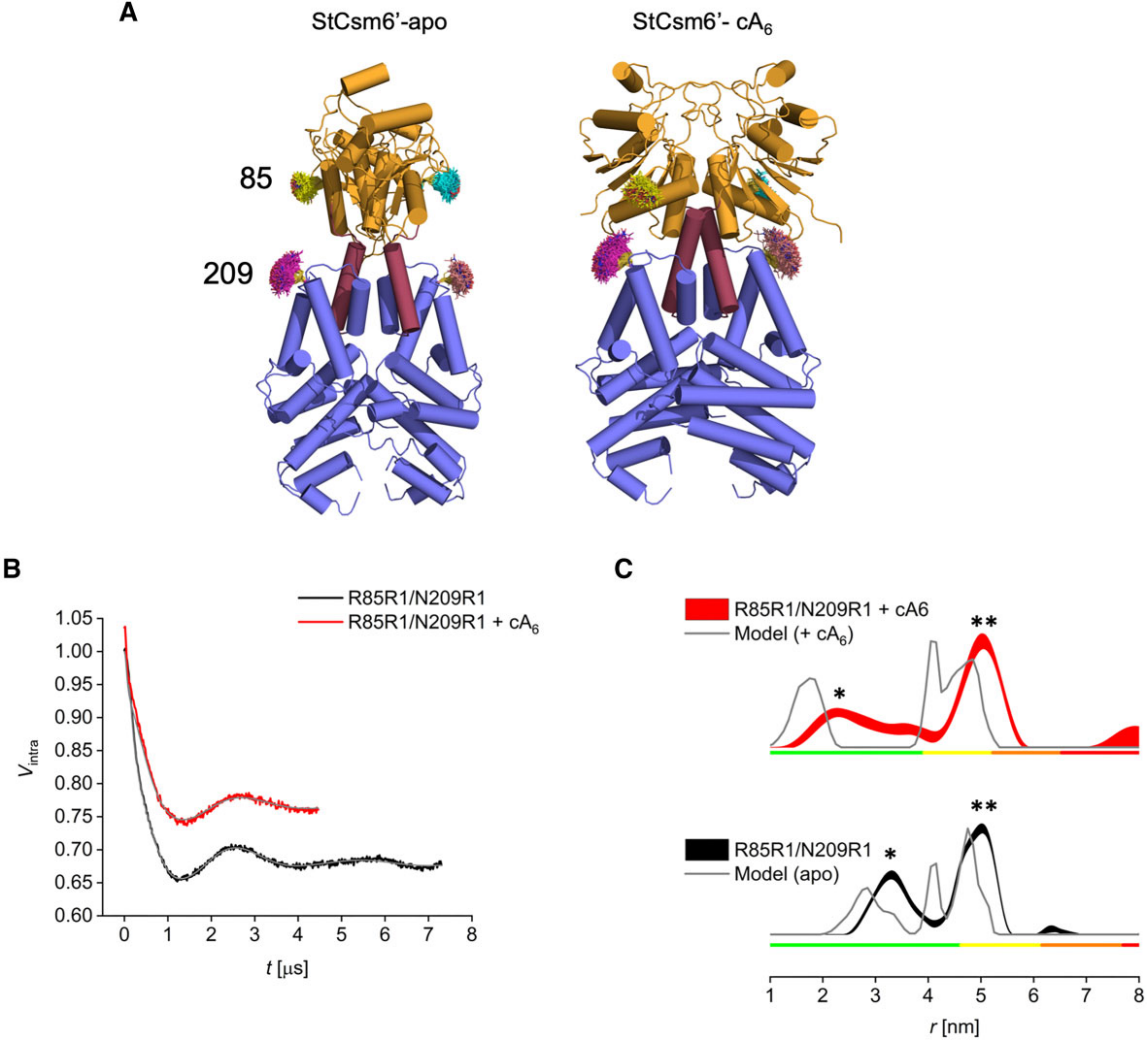
EPR data for StCsm6’ variant R85R1/N209R1 in the presence and absence of cA_6_. (**A**) Cartoon representation of the secondary structure elements of apo StCsm6’ (left) and in complex with cA_6_ (right), with colouring as described in Figure 4, showing the position and predicted MTSL rotamers for StCsm6’ D85R1/N209R1. (**B**) Background-corrected traces without (black) or with (red) addition of cA_6_ with fits (grey). (**C**) Distance distributions (shown as 95% confidence bands without (black) or with (red) addition of cA_6_) and modelled distributions based on the corresponding crystal structures (grey). Colour bars indicate reliability ranges (green: shape reliable; yellow: mean and width reliable; orange: mean reliable). Peak * corresponds to the distance between D85R1 and N209R1 in different monomers of the StCsm6’ dimer, which changes in the absence or presence of cA_6_. Peak ** corresponds to the overlapping distances between D85R1 in each monomer, N209R1 in each monomer, and D85R1 and N209R1 in the same monomer of StCsm6’, which are the same in the absence or presence of cA_6_.

To further validate the large-scale conformational transition between the apo and cA_6_ bound states of StCsm6’ observed in the crystal structures, pulse dipolar electron paramagnetic resonance spectroscopy (PDS) was employed (61). PDS gives access to ensemble distance distributions between paramagnetic spin labels in frozen solution and has been successfully utilized to probe conformational flexibility and ligand-induced structural changes in soluble and membrane proteins. A straightforward approach introduces two cysteines at the desired sites *via* site directed mutagenesis and post-translationally modifies them by site-specific spin-labelling with thiol specific nitroxide reagents yielding labelled side-chains (i.e. the spin-bearing residue R1 in the present case). As StCsm6’ is a homo-dimer, introduction of a single cysteine would yield a protein complex containing two spin labels. However, modelling revealed that in the dimer only minimal distance changes between any two identical amino acid residues in the two monomers are expected upon cA_6_ binding. Thus, double cysteine mutants were designed for which significant distance changes could be predicted (Figure 5A). While these result in multi-spin systems that are known to complicate analysis, this can be dealt with entirely on a post-processing level for up to four spins under our experimental conditions (65).

A double cysteine mutant of StCsm6’ at positions 85 and 209 was produced and spin-labelled with good yields (>85%) (Supplementary Figure S2). For R85R1/N209R1 in the apo structure of StCsm6’, a multimodal distance distribution was predicted with the shortest population centred around 3.0 nm and further intensity from 4.0 to 5.5 nm (Figure 5B, C; Supplementary Figure S10). While this latter population remains unchanged upon addition of cA_6_, the shorter distance is predicted to significantly reduce to 1.0–2.0 nm, which is well into the lower limit of PDS. Frozen solution CW EPR displayed dipolar broadening and the PDS echo decay was significantly accelerated, both consistent with the presence of a short distance (generally the lower limit of our PDS methodology lies at 1.5–1.8 nm). Despite the spin label distances in both states exceeding the models and hinting at a more expanded state in solution, the increase of short distance distributions was very clear in the PDS data. Results on an additional double cysteine mutant of StCsm6’, D88R1/N209R1, confirm this finding (Supplementary Figure S11). These data thus support the observation of the cA_6_-mediated conformational change observed in the crystallographic study, indicating the structures obtained *in crystallo* are consistent with those in solution.

### Mutagenesis of key interface residue D80 blocks activation of StCsm6’

**Figure 6.**
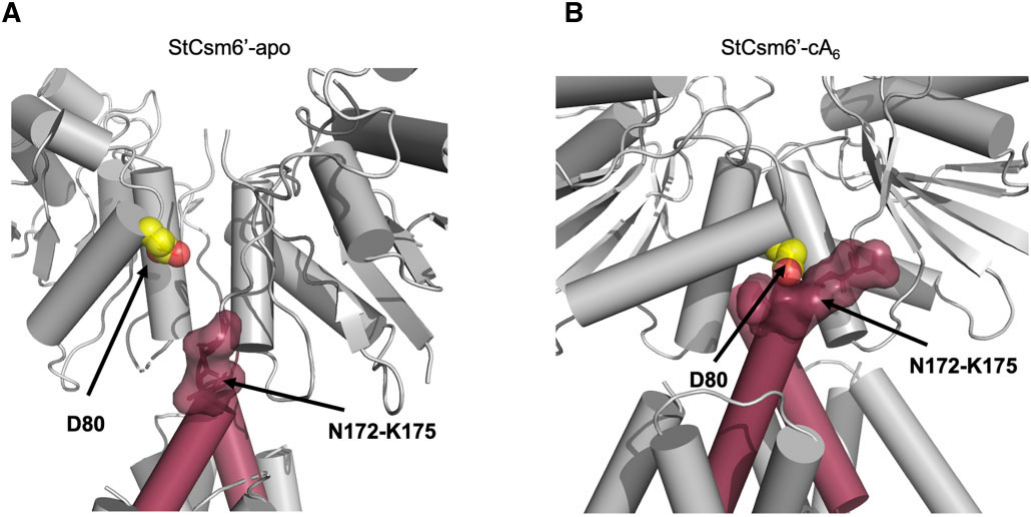
The role played by D80 in StCsm6’ upon cA_6_ binding. Cartoon representation of (**A**) apo StCsm6’ and (**B**) StCsm6’ in complex with cA_6_, showing key interactions between the CARF domain (grey) and an α-helix in the 6H domain (burgundy) is important for the conformational change brought about upon binding of cA_6_. In particular, D80 (shown as spheres, with carbon atoms in yellow and oxygen in red) in the CARF domain moves significantly upon binding of cA_6_ to come into close proximity with residues 172–175 (modelled in surface representation) at the top of the key α-helix in the 6H domain.

To test the hypothesis that the conformational change observed upon cA_6_ binding to StCsm6’ is required for activation, we mutated residue D80 to a bulkier tyrosine residue. In the apo StCsm6’ structure, D80 sits in a solvent exposed position in the CARF domain (Figure 6A; Supplementary Figure S7), but upon binding of cA_6_ the rotation of the CARF domain brings D80 into close contact with the residues at the top of the 6H domain (residues 172–175) (Figure 6B). We hypothesized there would be insufficient room to accommodate a bulkier tyrosine side chain in place of the aspartate, and thus would block the conformational change required for activation. The D80Y variant was expressed and purified as described for the wild type protein, but was almost completely inactive in a cA_6_ dependent RNase assay (Figure 3A). This is consistent with a model whereby cA_6_-mediated conformational changes, transmitted from the CARF domains to the HEPN domains, occurs through precise movement of the 6H domains, confirming their role in activation of the enzyme.

## DISCUSSION

Csm6 family proteins have attracted considerable interest as an exemplar of activatable, non-specific nucleases linked to type III CRISPR systems. They display minimal activity in the absence of the cOA effector (29), but once activated function as efficient ribonucleases that degrade both viral and host RNA (5,9,66). These properties have been utilized to develop a range of sensitive new diagnostic assays (19,67). The key question of how cOA binding at the CARF domain results in activation of the ribonuclease active site in the HEPN domain has remained unresolved, with only modest changes detected by crystallography when the two states are accessible to crystallization. Here, we have focussed on the cA_6_ activated family of Csm6 enzymes, which are broadly distributed in bacteria (1,2,8). These comprise a distinct sub-family of the structurally diverse Csm6/Csx1 family, whose members are most commonly activated by cA_4_. We succeeded in crystallizing apo- and cA_6_ bound *S. thermophilus* Csm6’, allowing a detailed comparison of the inactive and activated states. This revealed a remarkable structural transition, where binding of cA_6_ to the CARF domains results in a tightening of the secondary structure elements concomitant with a 58° rotation of the CARF domains relative to the HEPN domains. This movement, transmitted through the 6H domain, allows the ‘jaws’ of the HEPN domains to open and causes a rearrangement of the catalytic residues to form a functional active site prior to catalysis. To check the structural rearrangements observed were not artefacts of crystallization, EPR spectroscopy studies were conducted on StCsm6’ in the absence and presence of cA_6_ in solution, which showed changes in distances between key atoms were consistent with the X-ray crystallographic structures.

The mechanics of opening the jaws of the HEPN domains upon cA_6_ binding suggests this plays an important regulatory role to control ssRNA binding and nuclease activity. Although there is no structure of RNA bound to the HEPN domain of a member of the Csm6/Csx1 family for direct comparison, it is plausible that there is insufficient space for ssRNA to bind in the ‘closed’ form of the HEPN domains. Upon binding of cA_6_ to the CARF domains, the structural transition opens the HEPN domains providing ssRNA easier access to the active site, as well as rearranging residues to form a functional active site poised for catalysis. The relative importance of these two factors for Csm6 activation is unclear, and it remains to be determined whether the HEPN dimer binds and cleaves a single molecule of ssRNA in a composite active site.

The observation of both cA_6_ and the cleaved A_3_ > P products in the active site of the CARF domains of StCsm6’ provides unique insights into catalysis. The cleavage presumably occurred during the co-crystallization of StCsm6’ with cA_6_, but the restrictions of crystal formation meant catalysis was slow, thus allowing both substrate and product to be observed. It is proposed that an activated 2′-OH group on a ribose in a cyclic oligoadenylate molecule undergoes in-line nucleophilic attack on the adjacent scissile phosphodiester bond, and in order for this to happen the angle formed between the 2′-OH, P and O should be close to 180° (21,24,28), in this case 172°. Like EiCsm6 (24), the 2′-OH group of ribose and the phosphate involved in cleavage form only two interactions with StCsm6’, and neither of them are sufficiently reactive to play a role in catalysis. This is highly suggestive that StCsm6’ does not assist cleavage by deprotonation of the nucleophile or protonation of the leaving group, and instead it is purely the conformation adopted by cA_6_ in the binding site that drives catalysis, which would be consistent with a function in slow auto-deactivation of the enzyme.

The individual CARF, 6H and HEPN domains of apo StCSm6’, StCSm6’ in complex with cA_6_, and EiCsm6 in complex with cFA_6_ are structurally very similar, even though in some cases the sequence identity is low. However, the structural alignments for the dimeric domains, and the monomeric or dimeric full length proteins, are often poor with high RMSDs. This highlights that although the overall structural fold and interactions made locally are conserved within domains, it is the global changes in the conformation and orientation of those domains that are key to the activation of StCsm6’ upon binding of cA_6_. Intriguingly, the structure of EiCsm6 in complex with cFA_6_ displays an 'intermediate state' between the apo StCsm6’ and StCsm6’ in complex with cA_6_ structures (Supplementary Figure S9). One caveat is that the EiCsm6 structure may have been influenced by the presence of a non-hydrolysable cA_6_ mimic bound to the CARF domains. It is likely that apo StCsm6' can access a range of conformations by rotating the CARF domains, but is unable to achieve a stable active conformation until the cA_6_ activator is bound. This possibility is reinforced by the observation that the D80Y variant, which cannot access the final conformation, is inactive. This mobility may explain why it has proven difficult to crystallize the apo form of StCsm6—something we achieved here with a degree of serendipity.

*Streptococcus thermophilus* is unusual in having two Csm6 orthologues (Csm6 and Csm6’), sharing 34% identity at the amino acid level (1). Each forms a homodimer and is activated by cA_6_, although Csm6 is activated by lower levels of cA_6_ than Csm6’ (1). Both degrade cA_6_ bound in the CARF domain ((25) and this work). The reason for their co-existence in *S. thermophilus* may relate to defence against anti-CRISPRs, as suggested previously (1), differing activation and deactivation kinetics to give a broader range of response to changing cA_6_ concentrations, or simply redundancy.

In summary, we have demonstrated a dramatic structural transition between the apo and cA_6_-bound forms of StCsm6’, which underlies the activation of nuclease activity upon binding cA_6_. These structural insights show the 6H domains, which connect the CARF and HEPN domains in StCsm6’, play a key role in facilitating a large rotational movement to open the ‘jaws’ of the HEPN domains and reorientate the active site.

## Supplementary Material

gkad739_Supplemental_Files

## Data Availability

Structure coordinates and raw X-ray data have been deposited in the Protein DataBank with accession codes 8PCW and 8PE3. CDA reports for the EPR data are available as Supplementary files. The EPR research data underpinning this publication can be accessed at https://doi.org/10.17630/dd5a8ddf-4178-4602-8d90-407955431f3f.

## Notes

Present address: Januka S. Athukoralage, Arc Institute, Palo Alto, California, USA.
