# Supplementary material for: Activation of Csm6 ribonuclease by cyclic nucleotide binding: in an emergency, twist to open
Source: Nucleic Acids Res. 2023 Sep 25;51(19):10590–605. doi: 10.1093/nar/gkad739 (PMC10702470; doi:10.1093/nar/gkad739)
Supplement: gkad739_Supplemental_Files [file gkad739_Supplemental_Files.zip › Supplementary Information.docx]

**Supplementary Information**

**Activation of Csm6 ribonuclease by cyclic nucleotide binding: in an emergency, twist to open**

Stuart McQuarrie^1#^, Januka S. Athukoralage^1†#^_,_ Stephen A. McMahon^1^, Shirley Graham^1^, Katrin Ackermann^2^, Bela E. Bode^2^, Malcolm F. White^1^* and Tracey M. Gloster^1^*

^1^ Biomedical Sciences Research Complex, School of Biology, University of St Andrews, North Haugh, St Andrews, Fife, KY16 9ST, UK.

^2^ Biomedical Sciences Research Complex, School of Chemistry, Centre of Magnetic Resonance, University of St Andrews, North Haugh, St Andrews, Fife, KY16 9ST, UK.

^†^ Present address: Arcadia Science, Emeryville, California, USA.

^#^ These authors contributed equally to the work.

* To whom correspondence should be addressed: email [mfw2@st-andrews.ac.uk](mailto:mfw2@st-andrews.ac.uk) or [tmg@st-andrews.ac.uk](mailto:tmg@st-andrews.ac.uk)

**Supplementary Figure 1.**

ATGGGTGTGTTAATTAGTGCCGTCGGGGATACAGACCCTTTTCGTAATTTTCACGATGGGGCCCTTATCCATATCGCCCGTAAGTATCGTCCGGAAAAAGTGATCCTGATTTTTAGCGAGCACACTGCTAAAAAGCAAGGTAATATCGAGAAAGCTCTTTTTAGCATTGCACCCAATTATGAGCCTGAGCTGATTATCCATGATCCTATCATTTCCGACAATGAAGTCCACATCTTCGATGTAATGTTTCAGCGCTTTTCGGATATTCTTCAAGAATACTATACTAAAGAAGACGAGTTTATCCTGAACTTATCAAGTGCGACGCCGCAGATTAAAAGCGCATTATTTGTTATTAACCGCTTAAATGGCATTAATGTGAAAGCAGTACAAGTTAGCAGTCCAGAACACGCTTCTAATGAGAATATCGGTCACGACAACGATGAAAATATCGATGAGTTGATCGAGGTCAACAAGGATAACAAAGTTAATTTTATCGACCGTACTATTGAGGATAACGCCGAGAAATTTAGTCAGGCATTGCTGAAAAAGACCGCCCGTGACTTCATCGAGAAATTTGATTACAAAGCCGCCCTTGACATCCTTGACCAGCTGAGTGACTTCCCGAACTTAAAGAGTGTACGTGAAGAAATCCGCGATGTTGTTAATTGTCTGTCGAAGCAGGATGTTCCCAAAGGGCTTCGTCACAAAAAGCTTAAAGAGGAGGAGCAGAAAATCCTTTCGGCATACCTTACAATCGAGTTACAGCGCGAGCGTGGGAATGTCTCGGAGAGTTTTATTCGTATTAAAAATTTAACTGAATTTATCCTTGAAGACTATATTAAGAAACGTTATCCCGGCCTGATTGACGAGTACTGCGAAGACATTCAAAAGTACTATCTTAGCCTGTTCGACTACTCAAAGCTGCTTAAAGCAACCAAGGAATTCAAATTAAAACGTACCATCGCCCCTATTATCGATATGAACTCATCTCGCAATAAAGTTGCCCACAGTCTTTCTCCTTTAGACTCTGATGCTGTCAAACAACTTGGAATTGCAATGAAAACCTTGAAGACGCTGGTCCGTGAGCAATACCATTTTTCTCAGAGCGATTTCAATTTCTATCAAGACTTGAACAAAATTCTTTTGACCAAGTTGAATTGA

**Supplementary Figure 1.** Codon optimised gene sequence encoding StCsm6’.

**Supplementary Figure 2.**

**
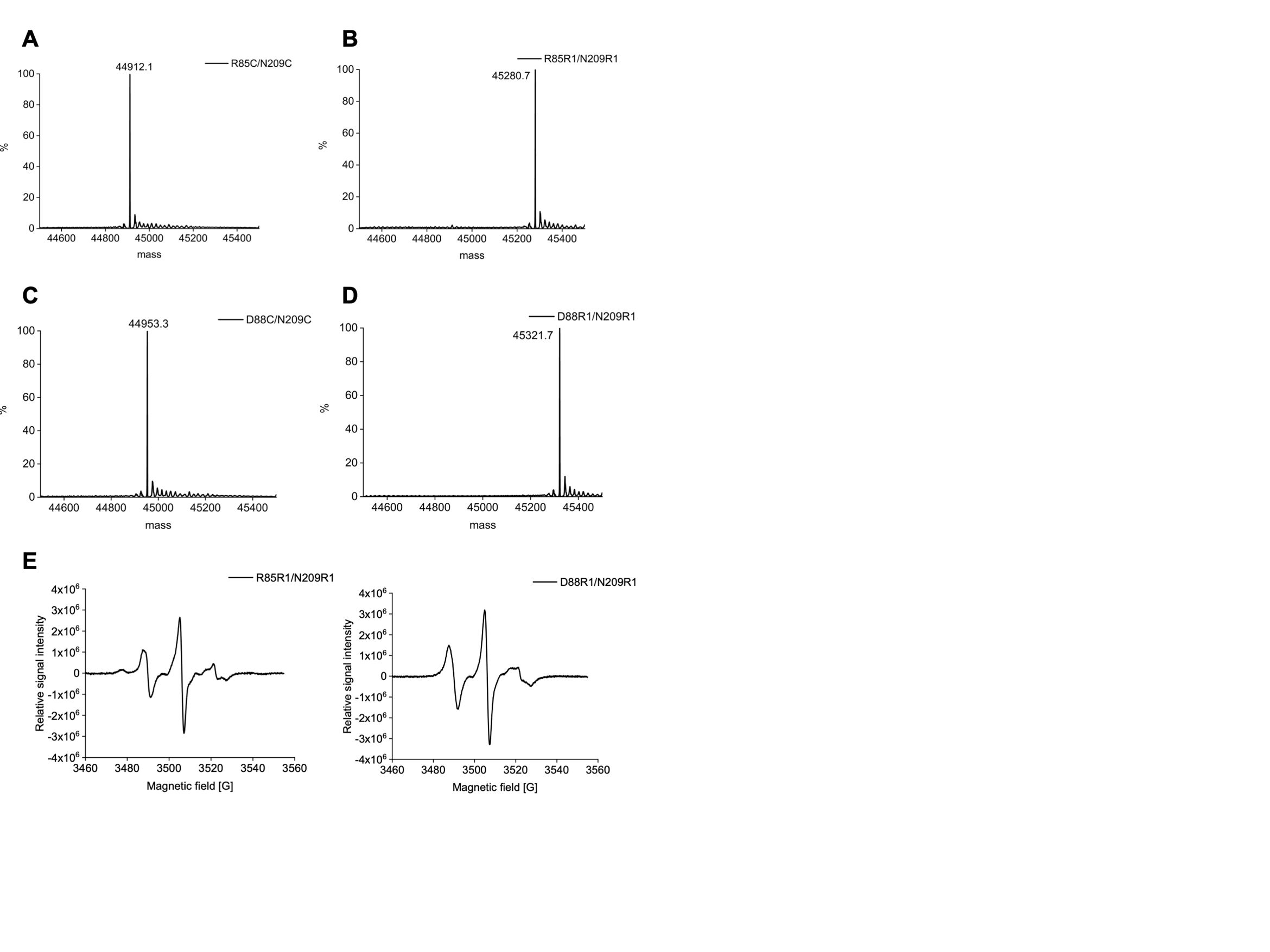
**

**Supplementary Figure 2.** Site directed spin labelling of StCsm6’. MaxEnt ESI spectrum (0.1 Da resolution) for **A**. unlabelled StCsm6’ R85C/N209C - the expected mass of 44,912 Da is observed; **B**. labelled StCsm6’ R85R1/N209R1 - the expected mass of 45,280 Da is observed; **C**. unlabelled StCsm6’ D88C/N209C - the expected mass of 44,953 Da is observed; **D**. labelled Csm6 D88R1/N209R1 - the expected mass of 45,321 Da is observed. **E**. Room temperature CW EPR spectra of StCsm6’ variants R85R1/N209R1 (left) and D88R1/N209R1 (right).

**Supplementary Figure 3.**

**
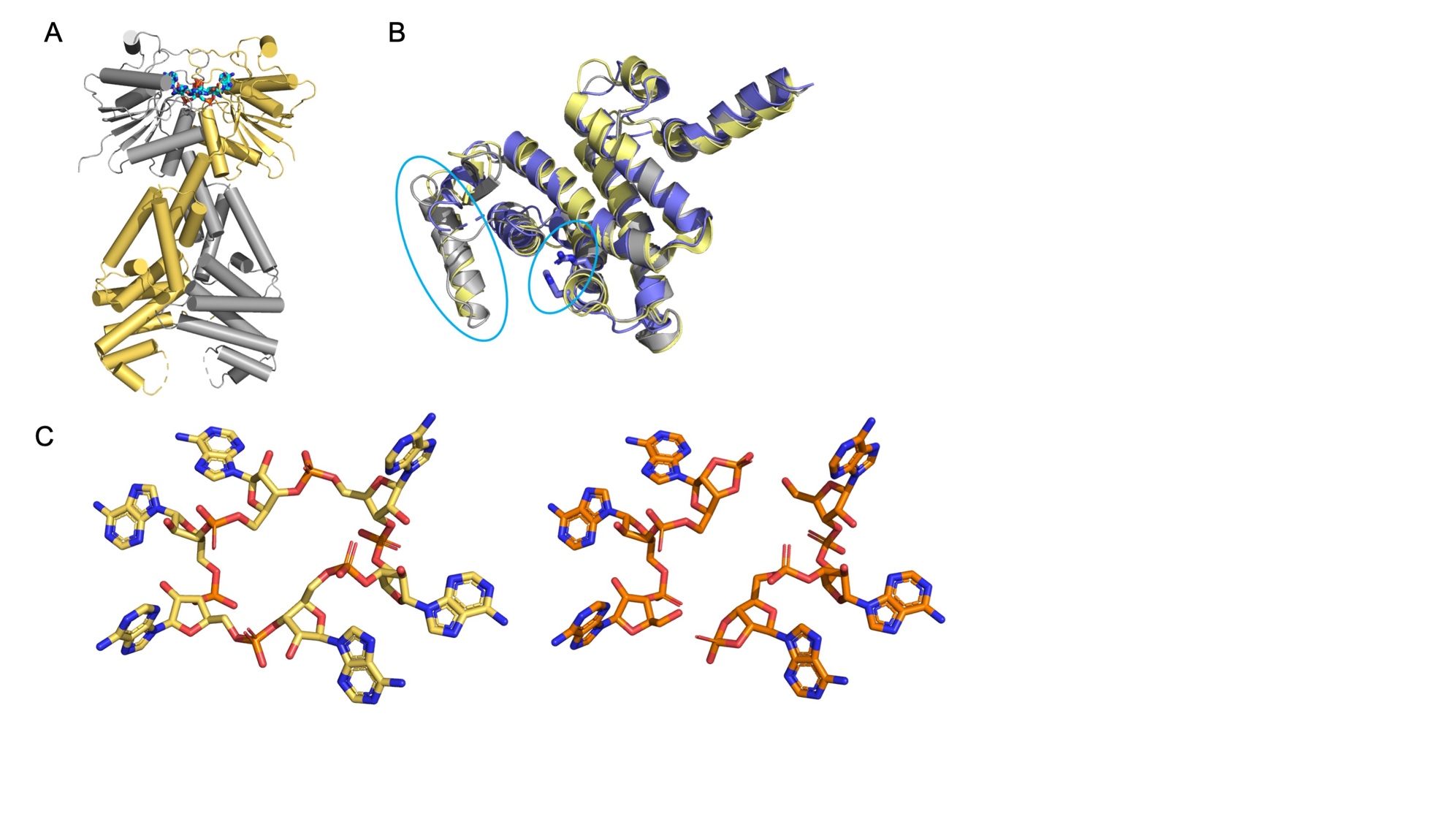
**

**Supplementary Figure 3. A.** Structure of StCsm6’ in complex with cA_6_. Each monomer in the dimer is coloured differently to illustrate the cross-over ‘X’ arrangement; cA_6_ is shown in cyan sticks. **B.** Superimposition of the HEPN domains of StCsm6’ in complex with cA_6_ (blue), EiCsm6 in complex with cFA_6_ (yellow) and SeCsm6 (grey). The extra secondary structure elements present in EiCsm6 and SeCsm6 are highlighted in the blue circle on the left, which is in close proximity to the active site R-X4-H motif highlighted in the blue circle on the right (with the catalytic residues, R331 and H336 from StCsm6’ shown in blue sticks). **C.** Stick representation of cA_6_ (left) and 2 molecules of A_3_>P (right), as observed in the binding site of the CARF domains. cA_6_ was modelled at an occupancy of 0.5 and both A_3_>P molecules at 0.5. Carbon atoms are shown in yellow for cA_6_ and orange for A_3_>P, with phosphorus in orange, nitrogen in blue, and oxygen in red.

**Supplementary Figure 4.**

**
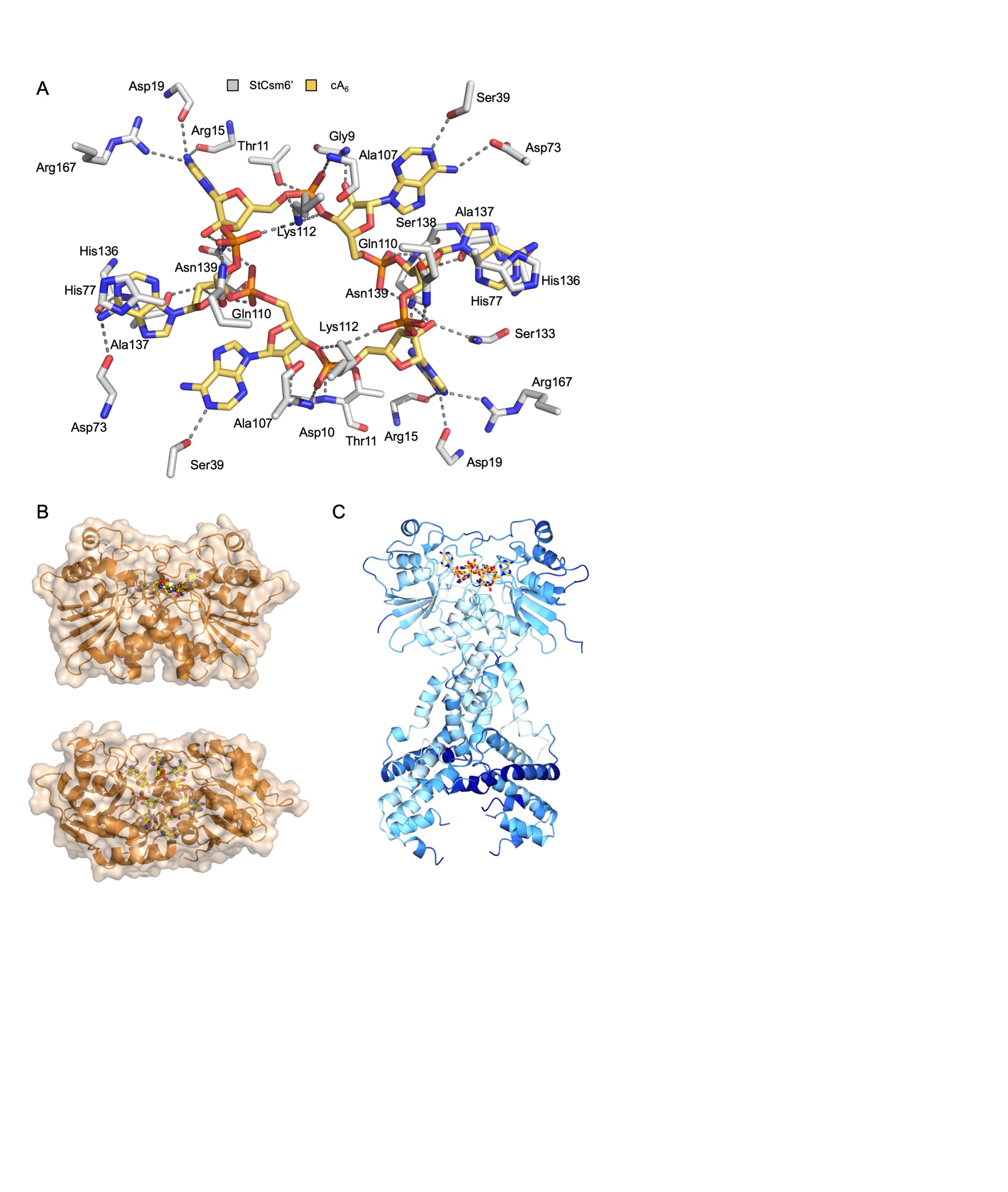
**

**Supplementary Figure 4. A.** Binding site of StCsm6’ in complex with cA_6_. Carbon atoms are shown in grey for StCsm6’ and in yellow for cA_6_, phosphorus in orange, nitrogen in blue, and oxygen in red. Hydrogen bonds are shown as grey dotted lines. **B.** Surface (with ribbon) representation of the CARF domains of StCsm6’ (orange) in complex with cA_6­_ (yellow sticks). Top panel shows ‘side’ view and bottom panel shows the ‘top down’ view of the binding site, illustrating that cA_6_ is completely enclosed. **C.** Ribbon representation of StCsm6’ in complex with cA_6_ (yellow sticks), coloured by temperature factor from white (low) to dark blue (high).

**Supplementary Figure 5.**

**
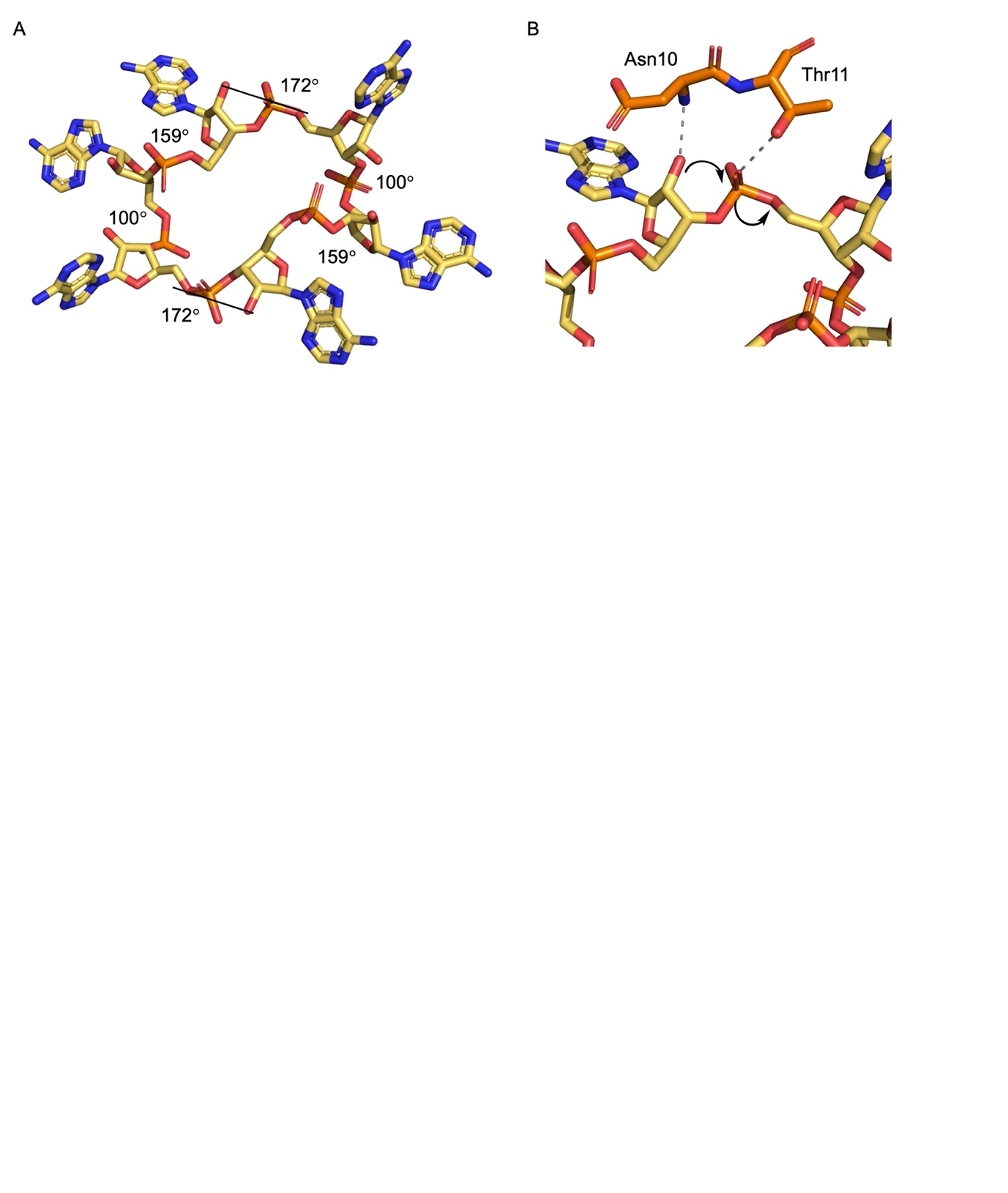
**

**Supplementary Figure 5. A.** Stick representation of cA_6_ as observed in the binding site of the CARF domains of StCsm6’. The angle between the 2’-OH of the ribose, phosphate and oxygen for each adenylate is shown. Cleavage takes place where this angle is close to 180°, as it facilitates in-line nucleophilic attack on the phosphodiester bond by the 2’-OH group of the ribose (and given the symmetry of cA_6_ occurs at identical, but opposite, positions of the ring). The black line illustrates where this angle is close to 180° in cA_6_, which agrees with position of the 2’,3’ cyclic phosphate moiety in the A_3_>P products observed (at half occupancy) in the binding site. Carbon atoms are shown in yellow for cA_6_, with phosphorus in orange, nitrogen in blue, and oxygen in red. **B.** Stick representation of part of cA_6_ (carbon atoms in yellow) as observed in the binding site of the CARF domains of StCsm6’ (carbon atoms in orange). Colours as described in **A**; hydrogen bonds are represented as grey dotted lines. The curly arrows illustrate the in-line nucleophilic attack by the 2’-OH group of the ribose.

**Supplementary Figure 6.**

**
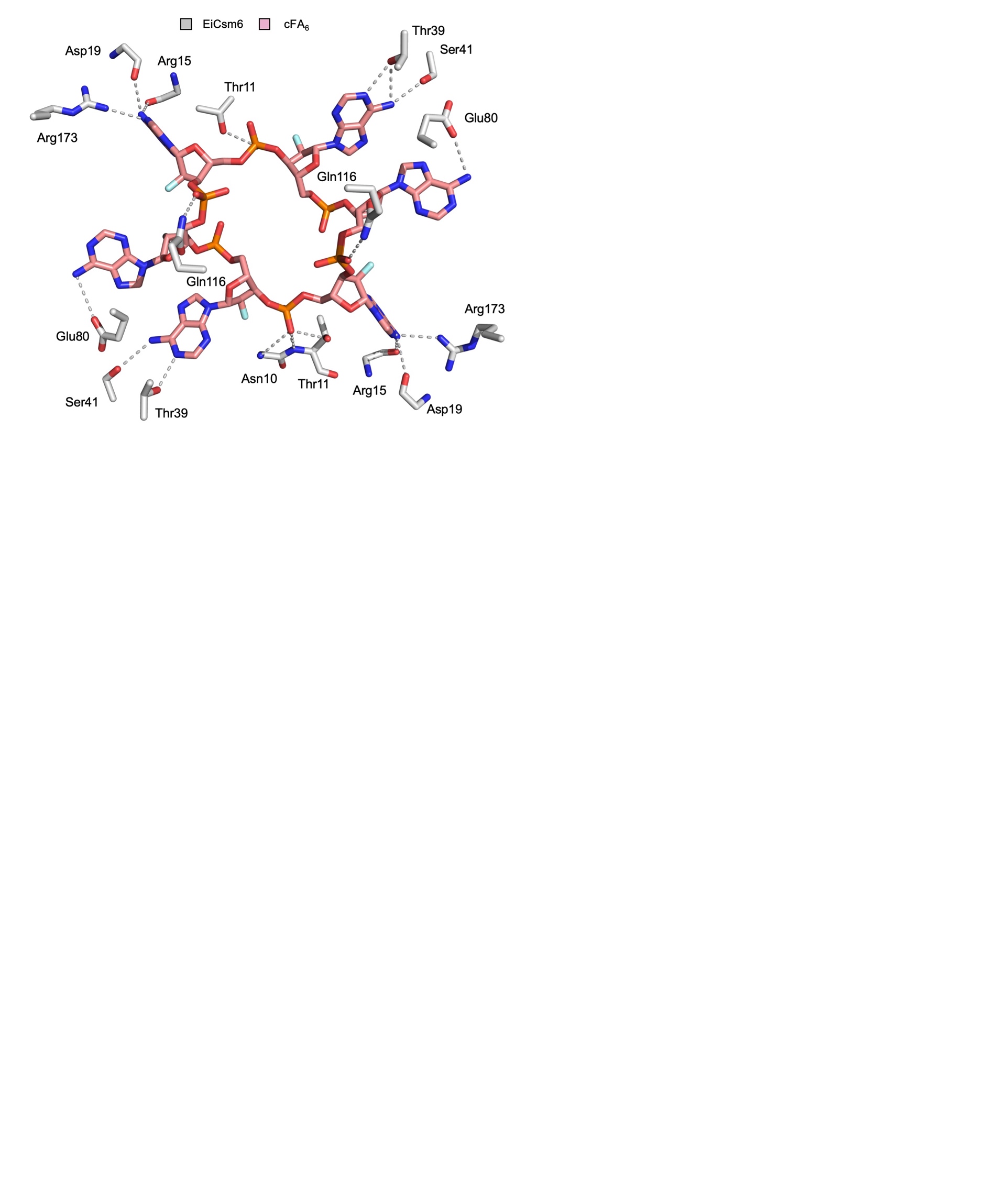
**

**Supplementary Figure 6.** Binding site of EiCsm6 in complex with cFA_6_. Carbon atoms are shown in grey for EiCsm6 and in pink for cA_6_, phosphorus in orange, nitrogen in blue, and oxygen in red. Hydrogen bonds are shown as grey dotted lines.

**Supplementary Figure 7.**


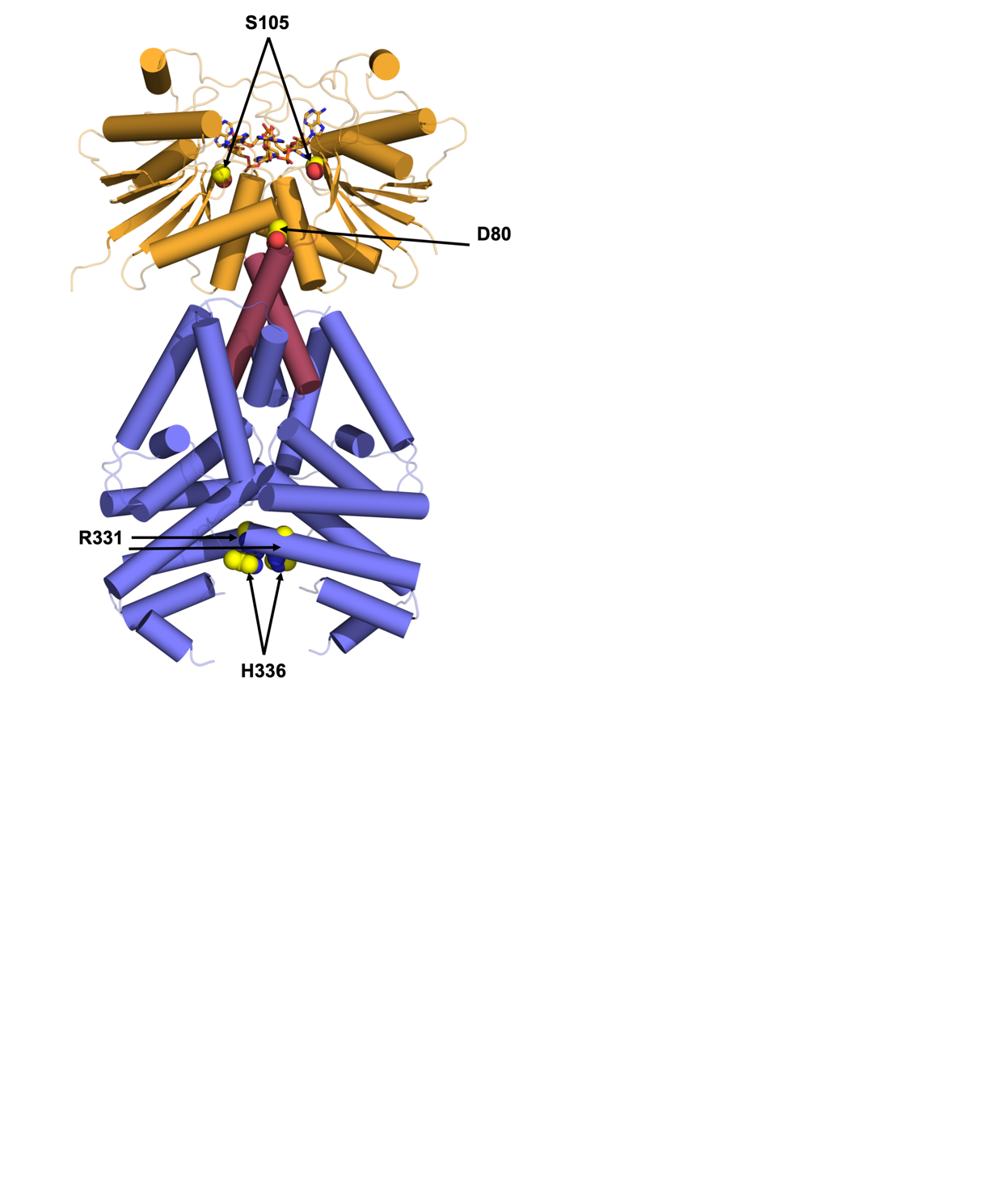


**Supplementary Figure 7.** Structure of StCsm6’ in complex with cA_6_, highlighting the position of mutations made as part of this study. Cartoon representation of the secondary structure elements of StCsm6’; the CARF domains are shown in orange, the 6H and HEPN domains in blue, and a key α-helix in the 6H domain in burgundy. The cA_6_ molecule is coloured in orange sticks, and the residues that were mutated (shown here as wild type residues) are highlighted in yellow spheres.

**Supplementary Figure 8.**


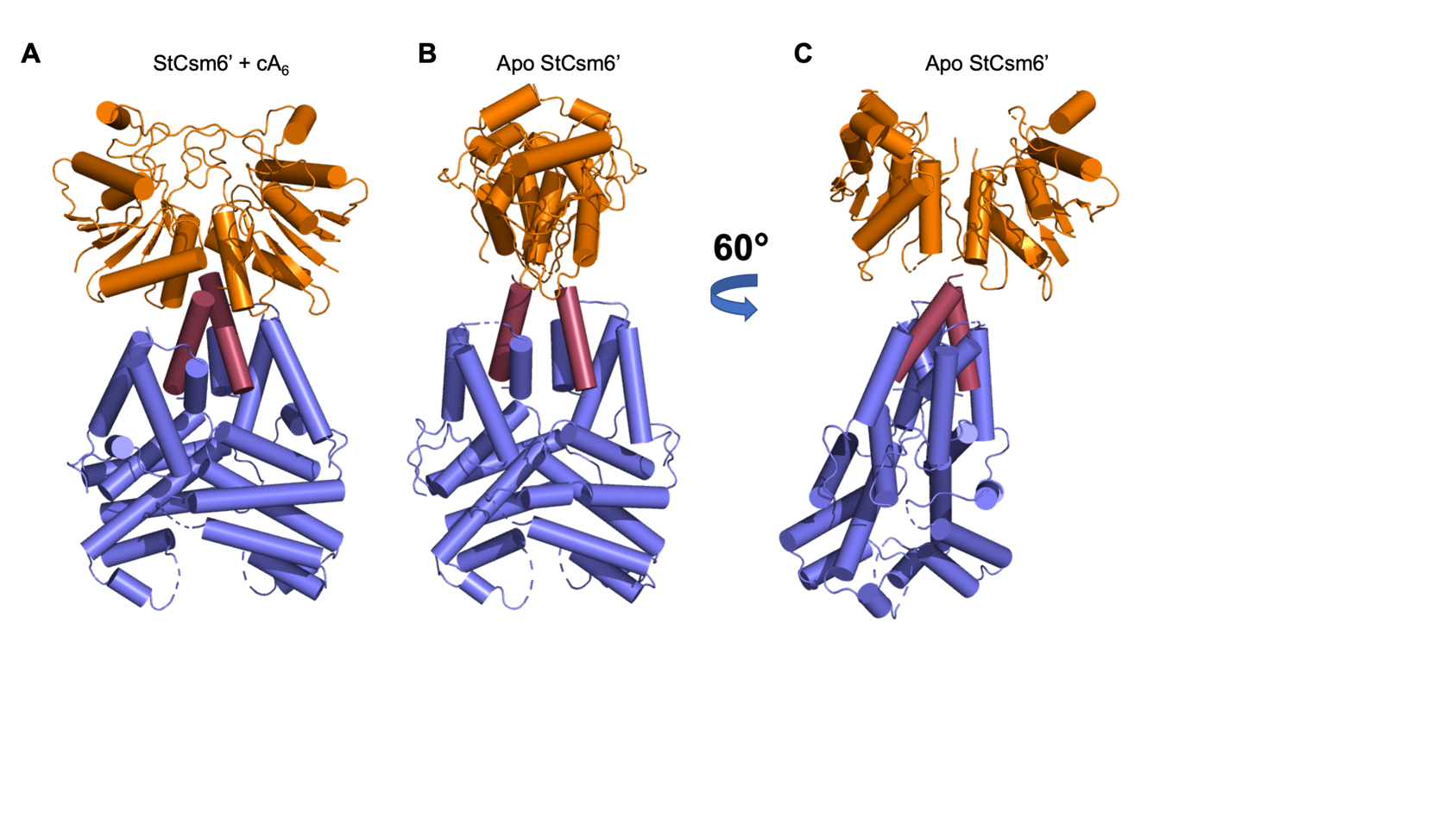


**Supplementary Figure 8. A.** Structure of StCsm6’ in complex with cA_6_ (ligand not shown). **B.** Structure of apo StCsm6’, which has been superimposed on the HEPN domains of StCsm6’ in complex with cA_6­_. **C.** Structure of apo StCsm6’ rotated 60° around the y axis relative to the structure in panel B. Note how the CARF domains are now in a similar orientation to those in the structure of StCsm6’ in complex with cA_6_ shown in panel A (with consideration that there is also movement of the secondary structure elements themselves which is not represented here). In all cases, cartoon representation of the secondary structure elements of StCsm6’; the CARF domains are shown in orange, the 6H and HEPN domains in blue, and a key α-helix in the 6H domain in burgundy.

**Supplementary Figure 9.**

**
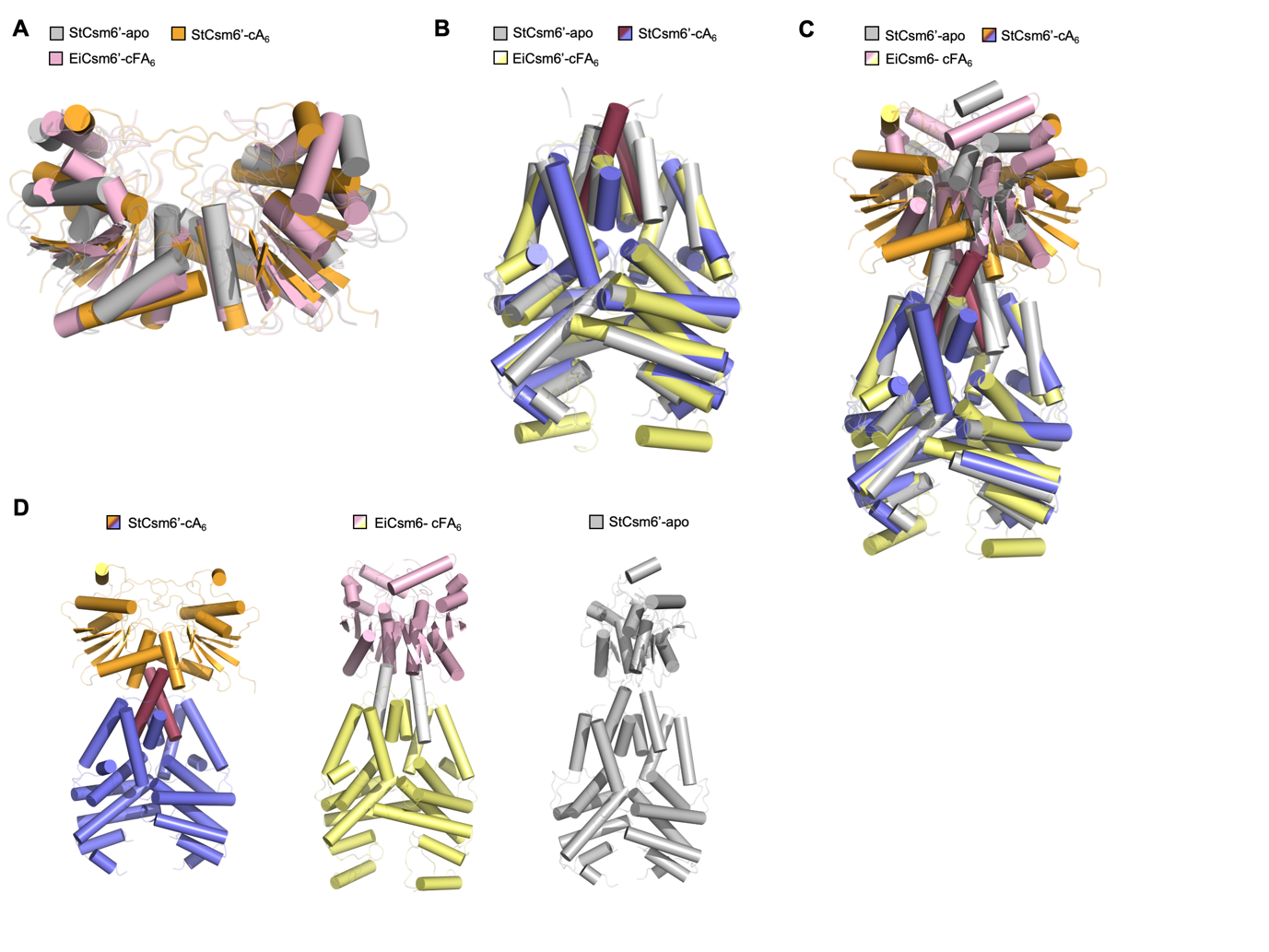
**

**Supplementary Figure 9. Comparison of apo StCsm6’, StCsm6’ in complex with cA_6_ and EiCsm6 in complex with cFA_6_ structures. A.** Cartoon representation of the superimposed (with the StCsm6’ in complex with cA_6­­­­_ structure as reference) secondary structure elements of the CARF domains of StCsm6’ in complex with cA_6_ (orange), apo StCsm6’ (grey), and EiCsm6 in complex with cFA_6_ (pink). **B.** Cartoon representation of the superimposed (with the StCsm6’ in complex with cA_6­­­­_ structure as reference) secondary structure elements of the HEPN domains of StCsm6’ in complex with cA_6_ (blue with key α-helix in the 6H domain in burgundy), apo StCsm6’ (grey), and EiCsm6 in complex with cFA_6_ (yellow with key α-helix in the 6H domain in white). **C**. Cartoon representation of the superimposed (with the HEPN domain of StCsm6’ in complex with cA_6­­­­_ structure as reference) secondary structure elements of the full length StCsm6’ in complex with cA_6_, apo StCsm6’, and EiCsm6 in complex with cFA_6_. Colouring as described in A and B. **D.** Cartoon representation of the secondary structure elements of the full length StCsm6’ in complex with cA_6_ (left), EiCsm6 in complex with cFA_6_ (middle) and apo StCsm6’ (right). These are in the same orientation as C (following superimposition of the structures with the HEPN domain of StCsm6’ in complex with cA_6­­­­_ as the reference). Colours as described in A and B.

**Supplementary Figure 10.**


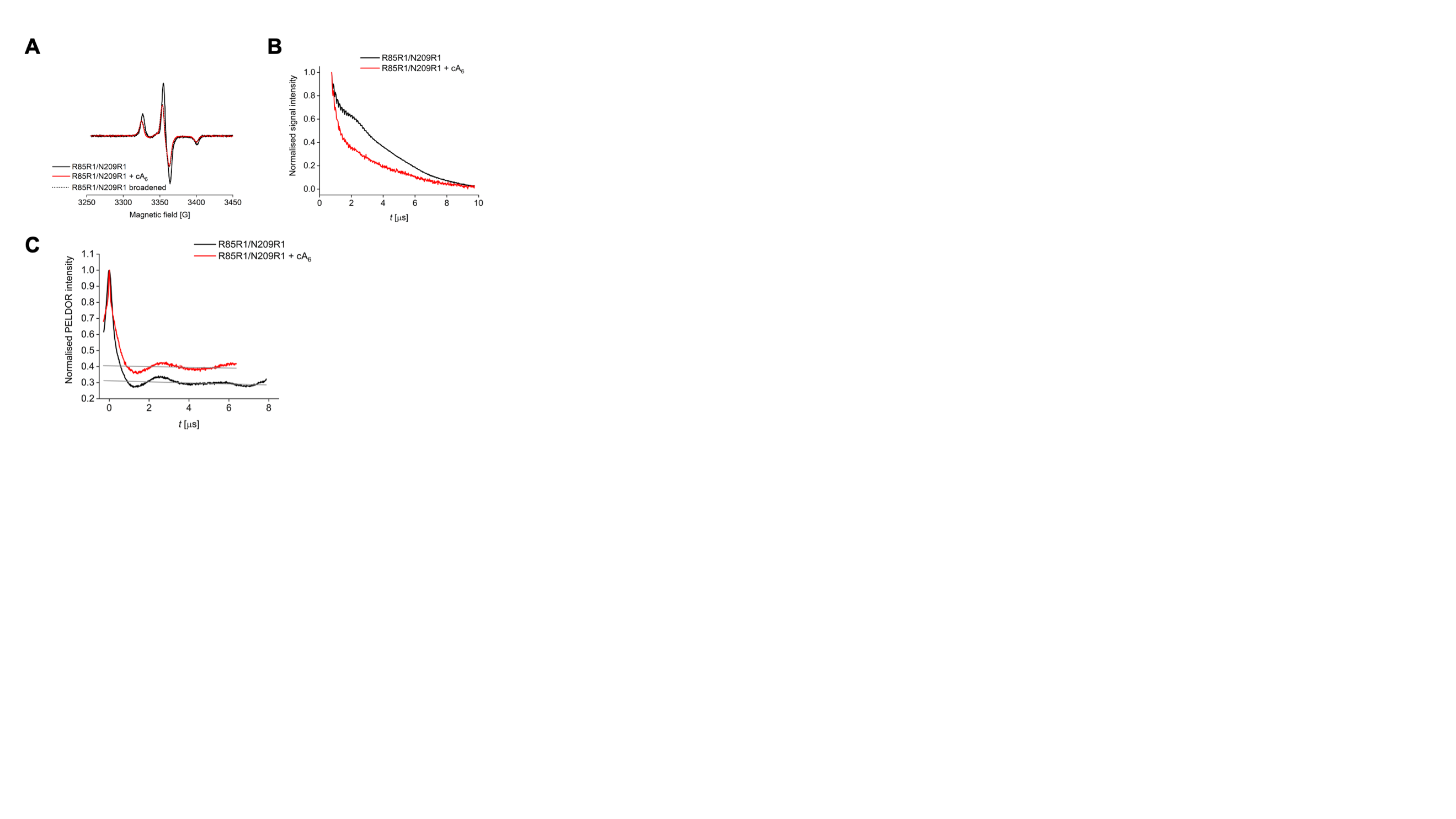


**Supplementary Figure 10.** EPR data for StCsm6’ variant R85R1/N209R1 without (black) or with (red) addition of cA_6_. **A.** Cryogenic CW EPR spectra. Minor broadening is observed corresponding to a Gaussian distance distribution centred at 1.1 nm with a standard deviation of 0.2 nm (dotted line). **B.** Refocused echo decays. Note the enhanced signal decay in presence of cA_6_. **C.** Raw PDS (PELDOR) data and 3-dimensional homogeneous background function (grey).

**Supplementary Figure 11.**


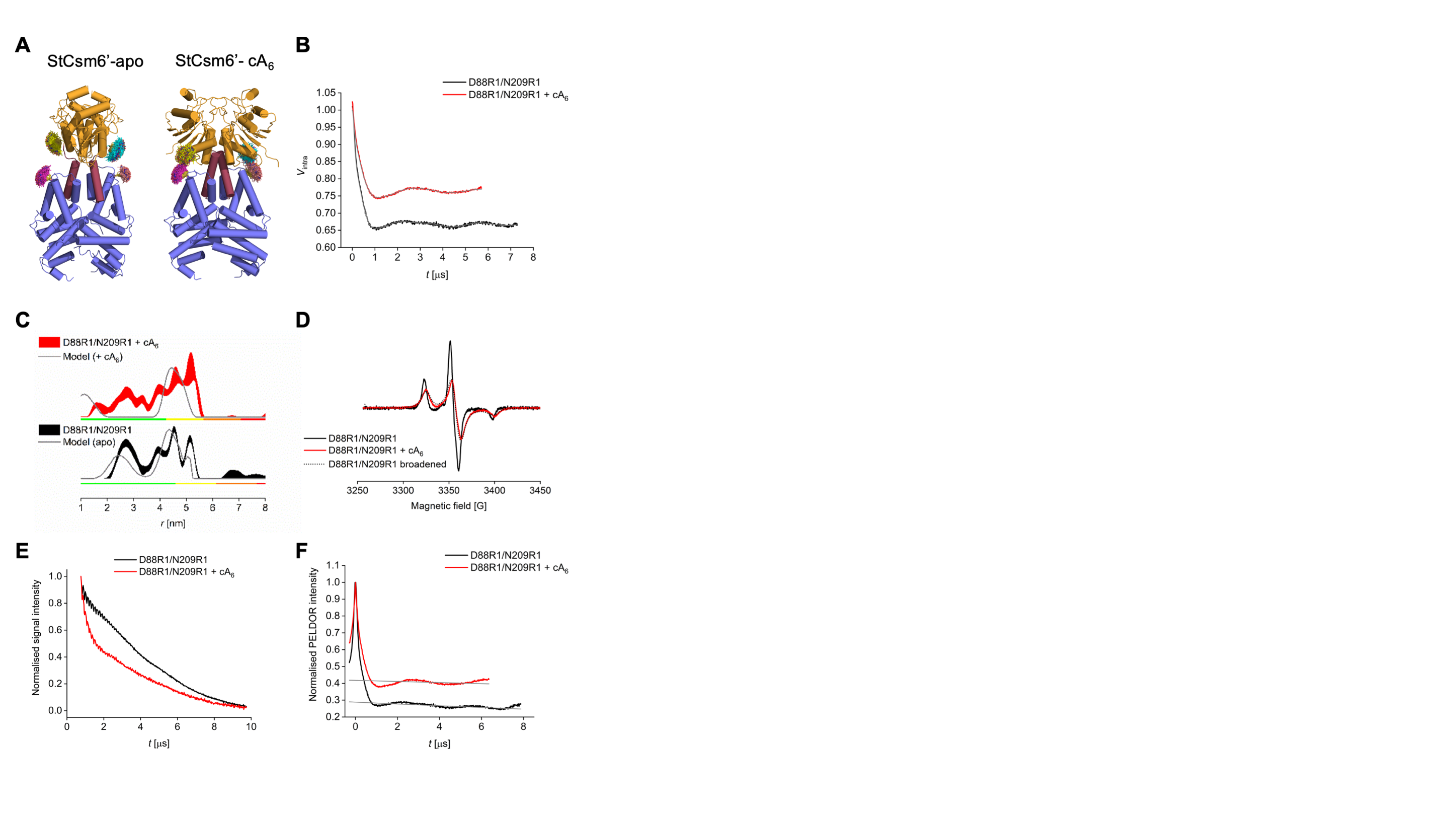


**Supplementary Figure 11.** EPR data for StCsm6’ variant D88R1/N209R1 without (black) or with (red) addition of cA_6_. **A.** Crystal structures with predicted MTSL rotamers for StCsm6’ D88R1/N209R1. **B.** Background-corrected traces with fits (grey). **C.** Distance distributions shown as 95% confidence band; overlaid are predicted distance distributions based on the corresponding crystal structures. Colour bars indicate reliability ranges (green: shape reliable; yellow: mean and width reliable; orange: mean reliable). **D.** Overlay of cryogenic CW EPR spectra. Substantial line broadening can be observed corresponding to a Gaussian distance distribution centred at 0.9 nm with a standard deviation of 0.35 nm (dotted line). **E.** Refocused echo decays. Note the enhanced signal decay in presence of cA_6_. **F.** Raw PDS (PELDOR) data, and 3-dimensional homogeneous background function (grey).

**Supplementary Table 1.** Data collection and refinement statistics of apo StCsm6’ and in complex with cA6.

|  | Apo-StCsm6’  (8PCW) | StCsm6’-cA_6_  (8PE3) | SeMet-  StCsm6’-cA_6_ |
| --- | --- | --- | --- |
| **Data collection** |  |  |  |
| Space group | *P*2_1_ 2_1_ 2_1_ | *P*4_3_ 2_1_ 2 | *P*4_3_ 2_1_ 2 |
| Cell dimensions |  |  |  |
| *a*, *b*, *c* (Å) | 88.65, 104.14, 144.48 | 99.91, 99.91, 286.53 | 100.11,100.11, 289.09 |
| α, β, γ (°) | 90, 90, 90 | 90, 90, 90 | 90, 90, 90 |
| Resolution (Å) | 29.50 – 3.54  (3.63 – 3.54) * | 94.3 – 1.96  (2.20 – 1.96) * | 32.8-2.61  (2.68 - 2.61) * |
| *R*_merge_ | 0.09 (1.34) * | 0.10 (1.35) * | 0.163 (3.57) * |
| *I* / s*I* | 9.4 (1.3) * | 14.8 (1.90) * | 19.7 (1.4) * |
| Completeness (%) | 95.2 (85.5) * | 95.1 (85.8) * | 99.9 (99.9) * |
| Redundancy | 3.8 (3.4) * | 13.1 (13.3) * | 36.2 (36.1) * |
| CC_1/2­_** | 1.0 (0.8) * | 1.0 (0.8) * | 1.0 (0.7) * |
| Wavelength (Å) | 0.9790 | 0.9795 | 0.9795 |
|  |  |  |  |
| **Refinement** |  |  |  |
| Resolution (Å) | 29.52 – 3.54 | 22.34 – 1.96 |  |
| No. reflections | 15131 | 54514 |  |
| *R*_work_ / *R*_free_ | 0.34 / 0.39 | 0.20 / 0.24 |  |
| No. atoms | 3,168 | 6441 |  |
| Protein | 3,168 | 6,043 |  |
| Ligand/ion | 0 | 132 |  |
| Water | 0 | 293 |  |
| *B*-factors (Å^2^) |  |  |  |
| Protein | 93.3 | 41.1 |  |
| Ligand/ion | n/a | 33.5 |  |
| Water | n/a | 40.0 |  |
| R.M.S.D.*** |  |  |  |
| Bond lengths (Å) | 0.0008 | 0.006 |  |
| Bond angles (°) | 0.23 | 0.87 |  |

* Values in parentheses are for the high resolution shell

** CC, correlation coefficient

*** R.M.S.D., root mean square deviation

**Supplementary Table 2.** RMSD values calculated by the DALI server for superimposition of full length StCsm6’ and its individual domains, in both the apo structure and in complex with cA6, in their monomeric and dimeric forms, against their nearest homologue in the PDB.

| **Structure used for search query** | | | **Comparison** | | **Alignment parameters** | | |
| --- | --- | --- | --- | --- | --- | --- | --- |
| **StCsm6’ apo/complex with cA_6_** | **Domain** | **Monomer/**  **Dimer** | **Searched against** | **PDB top hit** | **RMSD^1^ (Å)** | **RMSD over no. Cα atoms** | **No. residues in search** |
| cA_6_ | FL^2^ | Monomer | PDB | EiCsm6^3^ | 6.6 | 369 | 386 |
| cA_6_ | CARF | Monomer | PDB | EiCsm6 | 2.1 | 162 | 173 |
| cA_6_ | 6H | Monomer | PDB | EiCsm6 | 1.8 | 65 | 65 |
| cA_6_ | HEPN | Monomer | PDB | EiCsm6  SeCsm6 | 1.9  1.7 | 141  141 | 146 |
| Apo | FL | Monomer | PDB | EiCsm6 | 2.6 | 353 | 386 |
| Apo | CARF | Monomer | PDB | EiCsm6 | 1.9 | 152 | 173 |
| Apo | 6H | Monomer | PDB | EiCsm6 | 1.9 | 64 | 65 |
| Apo | HEPN | Monomer | PDB | EiCsm6 | 2.2 | 136 | 146 |
| cA_6_ | FL | Dimer | PDB | EiCsm6 | 2.6^4^ | 353 | 772 |
| cA_6_ | CARF | Dimer | PDB | EiCsm6 | 6.5 | 178 | 346 |
| cA_6_ | 6H | Dimer | PDB | EiCsm6 | 5.2 | 80 | 130 |
| cA_6_ | HEPN | Dimer | PDB | EiCsm6  SeCsm6 | 2.2  1.7 | 139  141 | 292 |
| Apo | FL | Dimer | PDB | EiCsm6  SeCsm6 | 4.8  1.9 | 238  210 | 772 |
| Apo | CARF | Dimer | PDB | EiCsm6 | 1.8 | 152 | 346 |
| Apo | 6H | Dimer | PDB | EiCsm6 | 3.4 | 69 | 130 |
| Apo | HEPN | Dimer | PDB | EiCsm6 | 2.0 | 136 | 292 |

1. RMSD, root mean square deviation

2. FL, full length

3. EiCsm6 corresponds to PDB code 6TUG; SeCsm6 corresponds to PDB code 5YJC

4. Green colouring highlights where the RMSD was calculated over significantly less Cα atoms than the number of residues in the search query, meaning the actual RMSD for the full length of the protein/domain will be much higher

**Supplementary Table 3.** RMSD values calculated by the DALI server for superimposition of full length StCsm6’ in complex with cA_6_, and its individual domains, in both their monomeric and dimeric forms, against the equivalent domains of apo StCsm6’.

| **Structure for pairwise alignment** | | | **Structure for pairwise alignment** | | | **Alignment parameters** | | |
| --- | --- | --- | --- | --- | --- | --- | --- | --- |
| **StCsm6’ + cA_6_** | **Domain** | **Monomer/Dimer** | **Apo**  **StCsm6’** | **Domain** | **Monomer**  **/Dimer** | **RMSD^1^ (Å)** | **RMSD over no. Cα atoms** | **No. residues in search** |
| cA_6_ | FL^2^ | Monomer | Apo | FL | Monomer | 7.7 | 327 | 386 |
| cA_6_ | FL | Dimer | Apo | FL | Dimer | 4.9^3^ | 441 | 772 |
| cA_6_ | CARF | Monomer | Apo | CARF | Monomer | 1.1 | 158 | 173 |
| cA_6_ | CARF | Dimer | Apo | CARF | Dimer | 4.6 | 316 | 346 |
| cA_6_ | 6H | Monomer | Apo | 6H | Monomer | 1.4 | 65 | 65 |
| cA_6_ | 6H | Dimer | Apo | 6H | Dimer | 3.8 | 128 | 130 |
| cA_6_ | HEPN | Monomer | Apo | HEPN | Monomer | 1.4 | 138 | 146 |
| cA_6_ | HEPN | Dimer | Apo | HEPN | Dimer | 2.8 | 274 | 292 |

1. RMSD, root mean square deviation

2. FL, full length

3. Green colouring highlights where the RMSD was calculated over significantly less Cα atoms than the number of residues in the search query, meaning the actual RMSD for the full length of the protein/domain will be much higher

**Supplementary Table 4.** ComparativeDeerAnalyzer key.

| **CDA report** | **StCsm6’ variant** |
| --- | --- |
| 210526_KAq172.6_DEER_comparative_DEER_analyzer_report | R85R1/N209R1 apo |
| 210703BEBQ50.2_DEER_comparative_DEER_analyzer_report | R85R1/N209R1 + cA_6_ |
| 210525_KAq172.3_DEER_comparative_DEER_analyzer_report | D88R1/N209R1 apo |
| 210705_BEBQ50.14_DEER_comparative_DEER_analyzer_report | D88R1/N209R1 + cA_6_ |
