# Supplementary material for: Activation of Csm6 ribonuclease by cyclic nucleotide binding: in an emergency, twist to open
Source: Nucleic Acids Res. 2023 Sep 25;51(19):10590–605. doi: 10.1093/nar/gkad739 (PMC10702470; doi:10.1093/nar/gkad739)
Supplement: gkad739_Supplemental_Files [file gkad739_Supplemental_Files.zip › Supplementary Movie 1 legend.docx]

**Supplementary Movie 1. Illustration of Csm6’ dynamics upon binding of cA_6_.**

(1) 0-5 s; Csm6’ dimer in complex with cA_6_ (Csm6’ monomers are shown in yellow and grey cartoon and cA_6_ is shown as spheres with carbon atoms in cyan).

(2) 6-16 s; Csm6’ in complex with cA_6_ (the CARF domains are shown in orange, 6H domains in burgundy and HEPN domains in blue cartoon, surface representation in grey, and cA_6_ is shown as spheres with carbon atoms in cyan).

(3) 17-22 s; CARF domains of Csm6’ (orange cartoon) in complex with cA_6_ (not shown) morphing to CARF domains of apo Csm6’.

(4) 23-40 s; apo Csm6’ (the CARF domains are shown in orange, 6H domains in burgundy and HEPN domains in blue cartoon, and surface representation in grey).

(5) 41-54 s; apo Csm6’ (the CARF domains are shown in orange, 6H domains in burgundy and HEPN domains in blue cartoon) morphing to Csm6’ in complex with cA_6_ (shown briefly as spheres with carbon atoms in cyan) and back again.

(6) 55-59 s; apo Csm6’ (the CARF domains are shown in orange, 6H domains in burgundy and HEPN domains in blue cartoon) morphing to Csm6’ in complex with cA_6_ (not shown) and back again. In the first part (55-59 s) apo Csm6’ is shown as grey cartoon and in the second part (60-64 s) Csm6’ in complex with cA_6_ is shown as grey cartoon.

(7) 65-74 s; apo Csm6’ (the CARF domains are shown in orange, 6H domains in burgundy and HEPN domains in blue cartoon, and surface representation in grey).

(8) 75-88 s; 6H (burgundy cartoon) and HEPN domains (blue cartoon) of apo Csm6’ morphing to Csm6’ in complex with cA_6_. The distance between F303 (last residue of the α-helix indicated) in each monomer is shown with a dashed line for apo Csm6’ (start of morph) and in complex with cA_6_ (end of morph).

(9) 89-95 s; Csm6’ in complex with cA_6_ (the CARF domains are shown in orange, 6H domains in burgundy and HEPN domains in blue cartoon, surface representation in grey, cA_6_ is not shown).

(10) 96-102 s; left, Csm6’ in complex with cA_6_ (the CARF domains are shown in orange, 6H domains in burgundy and HEPN domains in blue cartoon, surface representation in grey, cA_6_ is not shown); right, apo Csm6’ in the same colours.
