# Supplementary material for: Activation of Csm6 ribonuclease by cyclic nucleotide binding: in an emergency, twist to open
Source: Nucleic Acids Res. 2023 Sep 25;51(19):10590–605. doi: 10.1093/nar/gkad739 (PMC10702470; doi:10.1093/nar/gkad739)
Supplement: gkad739_Supplemental_Files [file gkad739_Supplemental_Files.zip › Supplementary Table 4 210526_KAq172.6_DEER_comparative_DEER_analyzer_report.pdf]

DEER analysis report on dataset 210526_KAq172.6_DEER

DEERNet Spinach SVN Rev 5662 and DeerLab 0.9.1 Tikhonov regularization

ComparativeDEERAnalyzer version 2.0

see: S. G. Worswick et al., DOI: 10.1126/sciadv.aat5218, L. Fabregas Ibanez et al., DOI: 10.5194/mr-1-209-2020

13-Dec-2022 10:24:30

1. Distance distributions

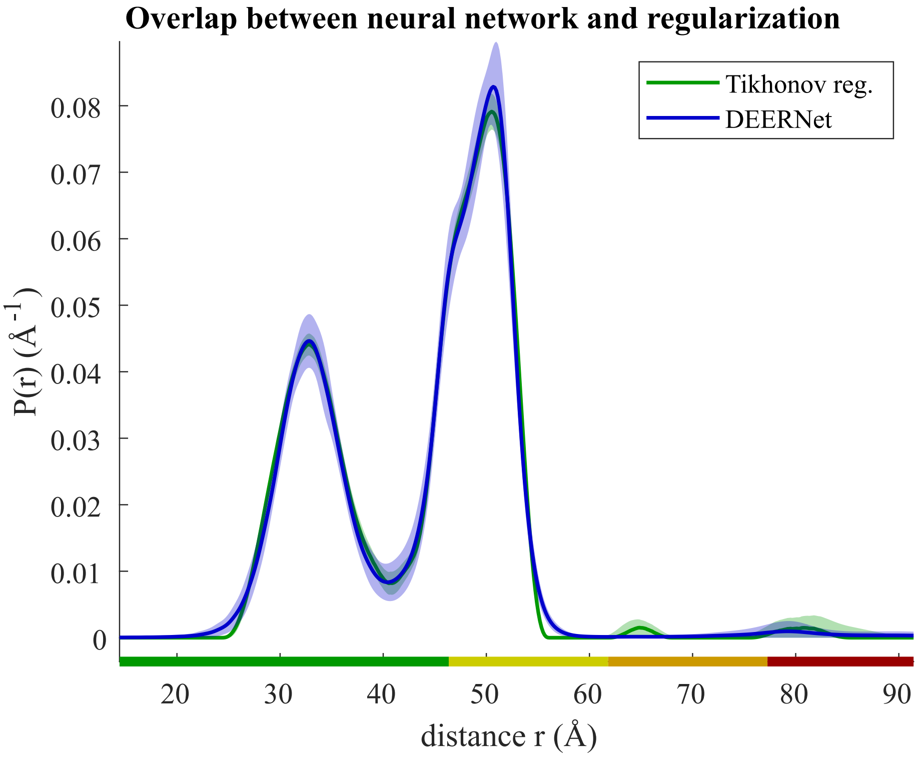

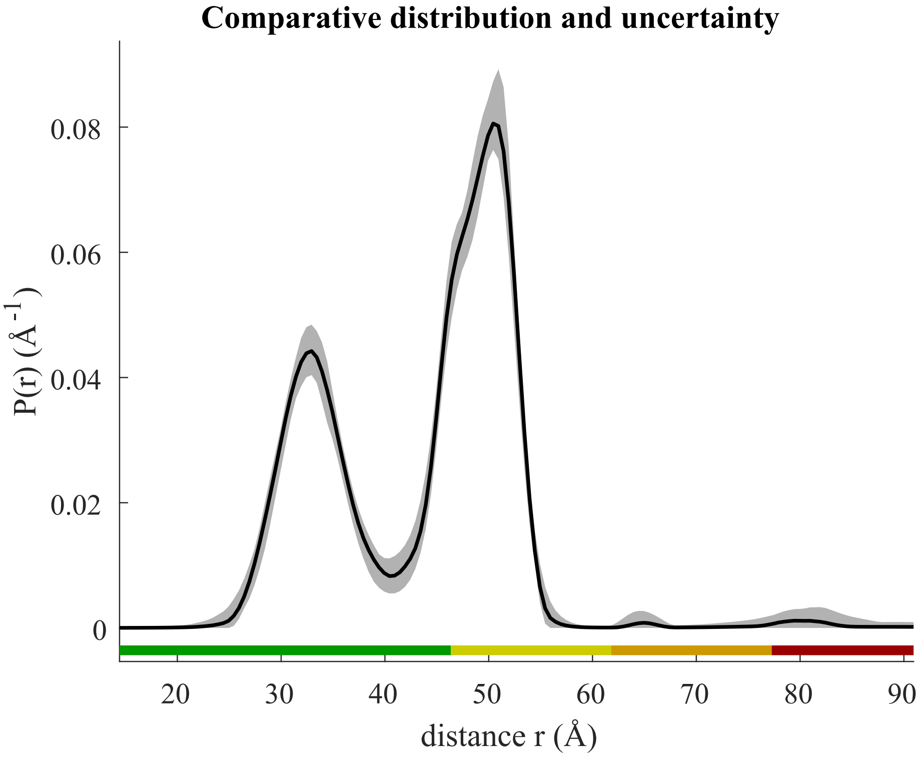

2. Fits of time-domain data

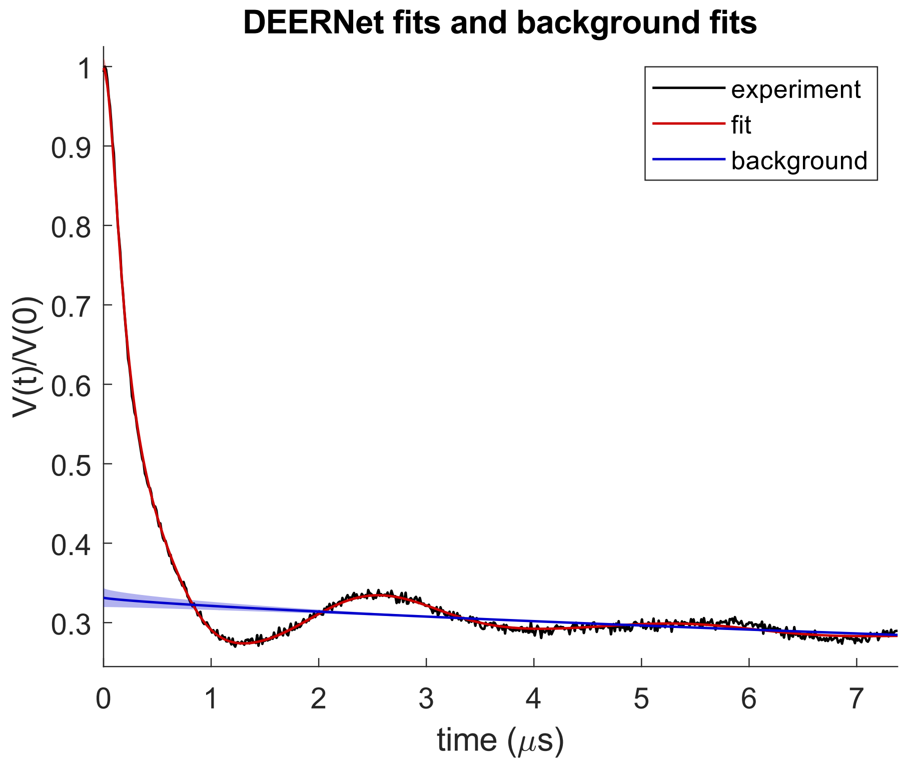

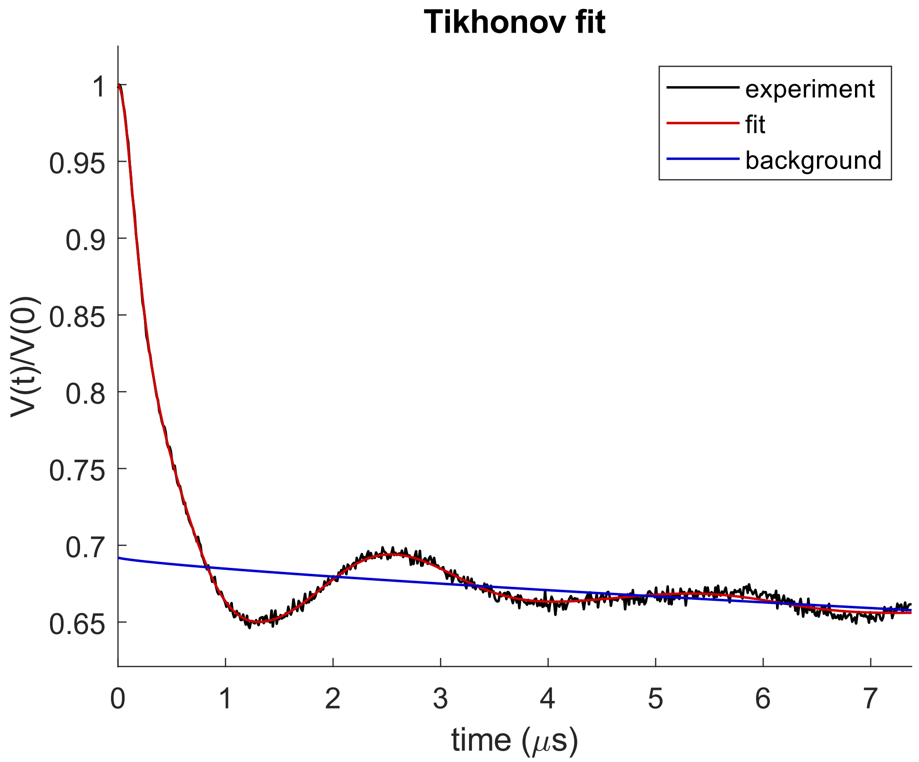

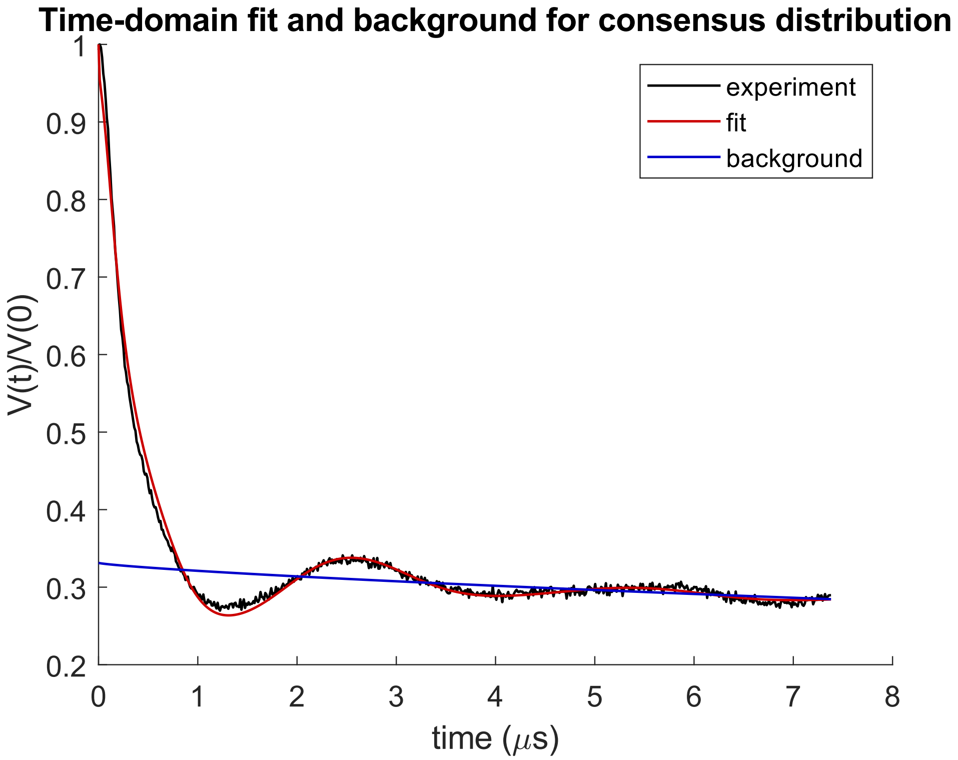
Time-domain fit and background for consensus distribution
experiment
fit
background
V(t)/V(0)
time (μs)
1
0.9
0.8
0.7
0.6
0.5
0.4
0.3
0.2
0
1
2
3
4
5
6
7
8

3. Experimental and processing parameters

Ghost suppression for a 4-spin system was applied.

Modulation depth: 0.668

Signal-to-noise ratio: 159.9 (w.r.t. modulation)

Noise estimates normalized to maximum signal

From imaginary part: 0.00447

From DEERNet fit: 0.00418

From Tikhonov fit: 0.00941

Zero time: 287 ns

Maximum time: 7380 ns

The last 6 % of the data was cut off

Time increment: 12 ns

Phase: 6.8 degree

Ensemble of 32 neural networks

Background separation by neural network

Background dimension: 3

Regularization parameter by best overlap with neural network solution

Regularization parameter used: 1.58

Reg. par. initial estimate by L-curve corner: 6.31

Overlap between DEERNet and regularization solutions: 0.968

Predicted overlap of consensus solution with ground truth: 0.83...1.00

Mean distance: 44.6 Å

Distance standard deviation: 7.7 Å

Full data set in Matlab format: C:\Users\ka44\Documents\OneDrive - University of St Andrews\StAndrews\Work\BEB\Projects\Csm6_MFW\Csm6_EPR\CDA2_DA2022_for_paper\210526_KAq172.6_DEER_comparative_DEER_analysis.mat

Distance distributions in text format: C:\Users\ka44\Documents\OneDrive - University of St Andrews\StAndrews\Work\BEB\Projects\Csm6_MFW\Csm6_EPR\CDA2_DA2022_for_paper\210526_KAq172.6_DEER_consensus_DEER_distribution.csv

Fit and background in text format: C:\Users\ka44\Documents\OneDrive - University of St Andrews\StAndrews\Work\BEB\Projects\Csm6_MFW\Csm6_EPR\CDA2_DA2022_for_paper\210526_KAq172.6_DEER_consensus_DEER_fit.csv

Metadata: C:\Users\ka44\Documents\OneDrive - University of St Andrews\StAndrews\Work\BEB\Projects\Csm6_MFW\Csm6_EPR\CDA2_DA2022_for_paper\210526_KAq172.6_DEER_comparative_DEER_meta_data.csv
